# “Proof *Under* Reasonable Doubt”: Ambiguity of the Norm Violation as Boundary Condition of Third-Party Punishment

**DOI:** 10.1177/01461672211067675

**Published:** 2022-02-01

**Authors:** Daniel Toribio-Flórez, Julia Saße, Anna Baumert

**Affiliations:** 1 Max Planck Institute for Research on Collective Goods, Bonn, Germany; 2 Technical University of Munich, Germany; 3 University of Wuppertal, Germany

**Keywords:** ambiguity, third party, costly punishment, norm violation, justice sensitivity

## Abstract

In six studies, we consistently observed *costly third-party punishment* (3PP) to decrease under *ambiguity of the norm violation*. Our research suggests that, under ambiguity, some people experience concerns about punishing unfairly. Those with higher (vs. lower) other-oriented justice sensitivity (Observer JS) reduced 3PP more pronouncedly (in Studies 1–3 and 4b, but not replicated in Studies 4–5). Moreover, those who decided to resolve the ambiguity (hence, removing the risk of punishing unfairly) exceeded the 3PP observed under no ambiguity (Study 4). However, we did not consistently observe these concerns about punishing unfairly to affect 3PP (Studies 4–5). We further considered whether people could use ambiguity as justification for remaining passive—thus, avoiding the costs of 3PP. We did not find conclusive evidence supporting this notion. Taken together, ambiguity entails a situational boundary of 3PP that sheds light on the prevalence of this behavior and, potentially, on its preceding decision-making.

Third-party punishment (3PP) can manifest itself in a wide range of phenomena, from confronting discrimination to speaking up against (cyber)bullying. It refers to sanctioning reactions against someone who violates a norm (i.e., perpetrator) by uninvolved bystanders. These reactions are considered desirable for the maintenance of social norms (Yamagishi, 1986); however, they usually entail costs for the third party, either physical (e.g., violence), social (e.g., ostracism), or economical (e.g., dismissal). Thus, the investigation of *costly* 3PP has raised special interest in multiple scientific fields (Krasnow et al., 2016; Lewisch et al., 2015; Riedl et al., 2012).

Researchers commonly investigate 3PP as the financially costly sanctioning of others who distribute monetary resources unequally between themselves and second parties. Empirical evidence from lab studies has shown that 50% to 60% of people engage in 3PP, with higher sanctions the more unequal the distributions are (e.g., Fehr & Fischbacher, 2004; Henrich et al., 2006). Critically, most of these studies provided decision-making settings with perfect situational information, allowing third parties to identify swiftly whether particular distributions constituted violations of fairness or equity norms. In real-life situations outside the lab, this is unlikely to occur. Individuals often receive noisy, incoherent, or incomplete information, which can create *ambiguity* about whether the perpetrator’s behavior actually adheres to or violates a norm. Resonating with this discrepancy, researchers have failed to observe comparable levels of 3PP in the field (e.g., Balafoutas et al., 2014; Brauer & Chekroun, 2005).

In line with theoretical models on bystander intervention (Baumert et al., 2013; Osswald et al., 2010), we assume that the interpretation of a norm violation as such is a necessary requirement for 3PP to occur. Therefore, we argue that ambiguity of a norm violation could constitute a pivotal boundary condition that hinders the decision of third parties to act against the norm violation. Previous research on 3PP has generally neglected the role of ambiguity of the norm violation. To our knowledge, only a recent set of studies (Jordan & Kteily, 2020, Studies 3a–b) has provided empirical evidence in this regard. In these studies, third parties showed less willingness to engage in indirect punishment (i.e., donation to an organization protesting against the perpetrator) against an ambiguous (vs. unambiguous) case of sexual harassment.

In the present research, we investigated how ambiguity of the norm violation influences direct 3PP and aimed to shed light on distinct motivations underlying this effect.

## Ambiguity of Norm Violations and 3PP

As an early critical step for 3PP, the interpretation of the situation has downstream effects on any further decision-making (Baumert et al., 2013; Osswald et al., 2010). If third parties access clear situational information, they can readily interpret the perpetrator’s behavior as a norm violation and then turn to ponder whether and how to react against it. Conversely, if the situational information is ambiguous, the interpretation of the norm violation should be hampered. At least, two psychological explanations make it plausible that ambiguity of the norm violation reduces 3PP.

First, third parties might refrain from punishing due to concerns of unfairly sanctioning someone who actually did not violate any norm. They might be aware that handling ambiguous information entails the risk of wrongly assuming that a norm violation has occurred when actually it did not (i.e., *type I error*; Grechenig et al., 2010). The motivation to avoid committing these type I errors could be fueled by anticipated feelings of guilt and reputational or moral concerns, as undeserved punishment might be negatively judged by others and by oneself.

Second, avoiding the costs of 3PP could be enticing to third parties. Individuals whose primary motivation is to avoid costs might use the ambiguity of the norm violation as a justification to remain passive. Supporting this reasoning, previous research has shown that people act less prosocially if the situation provides a justification for it (e.g., Dana et al., 2007). For instance, in a “dictator game”, researchers concealed how much money the recipient would actually receive from participants playing as dictators. In this setting, where uncertainty masked the dictator’s decision, participants were more likely to choose the option that was more beneficial to them. Furthermore, when researchers gave them the opportunity to reveal the concealed information, some participants deliberately avoided doing so. In light of these results, researchers suggested that some people might exploit situational ambiguity as “moral wiggle room,” which allows them to hide or justify selfish motives (Dana et al., 2007). We propose that this explanation could apply to 3PP as well. If a norm violation is ambiguous, some third parties who would punish under no ambiguity may use the situational ambiguity to justify their passiveness, thereby avoiding own costs (Kriss et al., 2016).

Taken together, we expect that ambiguity of the norm violation reduces 3PP because ambiguity introduces a risk of punishing unfairly and a potential justification to avoid incurring costs. To investigate these two underlying mechanisms, we employed two approaches: the examination of (a) interindividual differences in justice sensitivity (JS) as moderator of the effect of ambiguity and (b) the inclination of third parties to resolve the ambiguity. Both approximations aimed to distinguish between those who, under ambiguity, remain passive due to the risk of punishing unfairly and those who do so to avoid own costs.

## JS as Moderator of Reactions to Ambiguity

People may differ in the extent to which they are susceptible to the effects of ambiguity. Specifically, dispositional justice concerns should relate to individual motivations to avoid committing injustice and accept own costs to restore justice. Thus, we investigated the moderating role of interindividual differences in JS.

JS is a multidimensional personality construct that captures the strength of cognitive, emotional, and behavioral reactions to perceived injustice (Baumert & Schmitt, 2016). Researchers have distinguished four facets of JS, according to the perspectives from which people can experience and react to injustice (Schmitt et al., 2010). The perspective of a *perpetrator* who inflicts injustice on others (i.e., Perpetrator JS) and the perspective of an uninvolved *observer* (i.e., Observer JS) are relevant for the present research. Both perspectives refer to sensitivity to injustice done to others, and their combination has been observed to predict third-party reactions to norm violations (Lotz, Baumert, et al., 2011; Niesta Kayser et al., 2010). Critically, they conceptually relate to the mechanisms discussed to underlie the predicted effect of ambiguity on 3PP.

Perpetrator JS captures concerns about committing any injustice oneself (Baumert & Schmitt, 2016). Hence, people with high Perpetrator JS should be particularly concerned about punishing unfairly because unjustified punishment would constitute an act of injustice in and of itself. Thus, when ambiguity increases the danger of misinterpreting the norm violation (i.e., committing a Type I error), individuals with high (vs. low) Perpetrator JS should be more hesitant to punish.

For its part, Observer JS predisposes individuals to perceive injustice and be motivated to act against it, out of genuine other-oriented concerns for justice (Baumert et al., 2011; Schmitt et al., 2010). Observer JS has been found to relate negatively to selfish behavior (Edele et al., 2013; Fetchenhauer & Huang, 2004), even when the situation excused acting selfishly (Lotz et al., 2013). Therefore, people with low (and not high) Observer JS should readily exploit the ambiguity of the norm violation, as justification to remain passive and avoid own costs.

## Resolving the Ambiguity and Its Underlying Motivations

If third parties faced an ambiguous norm violation, gaining information that resolves the ambiguity would allow for informing the decision about whether or not to punish. Especially, third parties who wanted to avoid the risk of punishing unfairly should resolve the ambiguity. This would alleviate their type I error concerns, and therefore, it should facilitate exerting 3PP if a norm violation had actually occurred.

Conversely, third parties whose main goal is to avoid incurring costs do not gain from resolving the ambiguity. On the contrary, some might find keeping the situation ambiguous beneficial to uphold a situational justification for remaining passive (Dana et al., 2007; Stüber, 2020).

We therefore examined whether third parties who decide to resolve the ambiguity do so to punish subsequently the potential norm violation and whether those who do not resolve the ambiguity actually remain passive.

## Research Overview

In six studies, we investigated the effect of ambiguity of the norm violation and its underlying mechanisms on 3PP. In Studies 1 and 2, we tested the main effect of ambiguity on 3PP and the moderating role of JS. Studies 4 and 4b examined whether and why third parties would resolve the ambiguity before deciding to punish. Studies 3 and 5 aimed to rule out potential confounds of the experimental manipulation used in the other studies.

In every study, we used the third-party punishment game (3PPG) as experimental paradigm (Fehr & Fischbacher, 2004). The 3PPG follows the structure of a dictator game to the extent that the dictator (Person A) can distribute an endowment with a passive recipient (Person B). A third party (Person C), unaffected by the dictator’s decision, is informed about the dictator’s distribution and can influence it by deducting coins from the dictator’s final payoff (henceforth, *punishment* of Person A). In our studies, Person C could simultaneously add coins to the recipient’s final payoff (henceforth, *compensation* of Person B; for results about compensation, see Supplemental Tables S1–S5 and Supplemental Figures S1–S2). The addition of compensation to the 3PPG aimed to counter experimental demand effects, potentially occurring in experiments where third parties can solely punish (Lotz, Okimoto, et al., 2011; Pedersen et al., 2018). Critically, both punishment and compensation were associated with known costs for Person C.

We experimentally manipulated ambiguity of the norm violation by providing Person C with perfect or imperfect information about the endowment of Person A. In the *no ambiguity* conditions, participants learnt the exact endowment of Person A. In the *ambiguity* conditions, the endowment of Person A was randomly determined. While Person A was informed about their exact endowment before making their respective decision, Person C only learned about the range of possible endowments. Therefore, for Person C, it was ambiguous whether Person A’s distribution was unequal or not. Note that, in the dictator game, people generally regard unequal distributions as norm violations (Krupka & Weber, 2013).

## Studies 1 to 2

First, we tested our main hypothesis, which held that ambiguity of the norm violation would reduce 3PP (H1). Furthermore, we examined whether JS moderated this effect. Specifically, we expected that, under *ambiguity* (vs. *no ambiguity*), 3PP would decrease more pronouncedly among third parties with high (vs. low) Perpetrator JS (H2a). We also predicted that, under *ambiguity* (vs. *no ambiguity*), 3PP would decrease more pronouncedly among those with low (vs. high) Observer JS (H2b). We preregistered these hypotheses for Study 1 (https://osf.io/ubnzm) and Study 2 (https://osf.io/etgq9). Informed by the results of Study 1, the preregistration of Study 2 additionally included the competing hypothesis that the expected decrease of 3PP under ambiguity would be more pronounced among third parties with high (vs. low) Observer JS (H2b).

### Method

#### Open practices

The data, analysis code, codebook, and materials of Studies 1 to 5 are accessible at https://osf.io/2q9vm/.

#### Design

In Studies 1 and 2, each participant played four rounds of the 3PPG. In two rounds (*no ambiguity*), participants, playing as Person C, learnt that Person A received a fixed endowment of 10 experimental currency units (ECUs). In the other two rounds (*ambiguity*), they learnt that Person A would receive a randomly determined endowment that ranged from 2 to 10 ECUs.

The reason behind having four rounds was that these studies included, besides ambiguity, a second within-subject factor to address a further, yet unrelated research question, namely, whether uncertain (vs. certain) costs affected 3PP, independently of the ambiguity of the norm violation. Two of the four rounds established a certain cost of 0.5 ECU for Person C (per 1 ECU that they wished to punish or compensate), whereas in the other two rounds, this cost was uncertain, as it randomly varied between 0.01 and 1 ECUs. However, this manipulation of cost uncertainty did not exert any influence on 3PP, nor did it moderate the effect of ambiguity (for details, see Supplemental Tables S6–S9).

#### Participants

Studies 1 and 2 were part of a larger research project and aspects unrelated to the research questions at hand determined the sample size. Yet, we conducted sensitivity analyses to determine the minimum effect size that our recruited samples allowed us to detect with 90% statistical power and alpha = .05 (see below).

##### Study 1

We recruited 165 participants and excluded data of one based on preregistered criteria, including self-reported careless response and response times, as recommended by Meade and Craig (2012). The final sample consisted of 164 undergraduate students from diverse disciplines (77% women, age range = 18–33 years, *M* = 22.79 years, *SD* = 2.92 years). The smallest effect size we could detect with this sample size was a standardized regression weight of β = .10. Participants could earn up to €10 in the 3PPG.^1^

##### Study 2

We recruited 228 participants and excluded data of two based on the same preregistered criteria as in Study 1. The final sample consisted of 226, mainly undergraduate students from diverse disciplines (73% women, age range = 18–68 years, *M* = 23.26 years, *SD* = 5.44 years). The smallest effect size we could detect with this sample size was a standardized regression weight of β = .074. Participants could earn up to €10 in the 3PPG.

#### Procedure

Participation was either in the lab or online. After providing informed consent, participants completed the JS inventory, presented among different personality questionnaires (see preregistration for all materials).

Then, participants played the 3PPG. They learned that their decisions would have real financial consequences for themselves and others. They played the different rounds of the 3PPG in a fixed sequential order—Round 1 and Round 2 (*no ambiguity*), Round 3 and Round 4 (*ambiguity*). Several comprehension questions ensured that participants understood the rules of the 3PPG and the manipulated elements of each round (e.g., “How many [ECUs] does Person A receive?” to tap into the ambiguity manipulation). When participants answered them incorrectly, we repeated the instructions.

In the 3PPG, we implemented a strategy vector method (e.g., Oxoby & McLeish, 2004). The advantage of this approach was to obtain the complete decisional profile of each individual. This included their decisions in each role (Persons A, B, and C) and, when deciding as Person C, their conditional decisions to different distributions from Person A, ranging from fair to extremely unfair. Thus, participants sequentially decided in the role of Persons A, B, and C. In the critical role of Person C, participants received an endowment of 10 ECUs. They made decisions that were conditional on seven different possible distributions from Person A (i.e., “Given that Person A transfers [0 to 6] ECUs to Person B, how many ECUs do you wish to *deduct* from Person A’s/*add* to Person B’s endowment?”). Participants could punish Person A between 0 and the maximum remaining amount of money that Person A would have after each distribution, given an initial endowment of 10 ECUs. In the *ambiguity* condition, Person C did not know what the initial endowment of Person A was. Consequently, the participants’ punishment decision could potentially exceed the actual remaining endowment of Person A. However, participants were informed that their punishment would only become effective until Person A’s endowment was reduced to 0 ECUs, while they would still incur costs for the punishment they decided to apply. At the end of the experiment, we randomly grouped participants into triads and assigned them to one of the three roles. We then calculated their payoff based on the decisions they made in one round selected at random and the corresponding decisions of the other two members of the triad (for complete instructions, see Methodology File).

#### Measures

##### 3PP

We computed a continuous measure of 3PP in each condition by summing up the amount deducted by Person C in those decisions that implied a reaction to an unequal split of a 10-ECU endowment by Person A (i.e., Person A sent [0, 1, 2, 3, or 4] ECUs to Person B). We excluded the decisions entailing a fair split (i.e., Person A sent [5 or 6] ECUs) under the assumption that these would not generally be perceived as norm violations (Krupka & Weber, 2013). If participants did not report more than one decision in a round, their data were not included in the analyses for that round.

##### JS

We assessed JS with the German *Justice Sensitivity Short Scales* (Baumert, Beierlein, et al., 2014), which include two items each for measuring Perpetrator JS (e.g., “I feel guilty when I enrich myself at the cost of others”) and Observer JS (e.g., “I am upset when someone is undeservingly worse off than others”). For descriptive results, see Table 1.

**Table 1.** Descriptive Statistics and Cronbach’s Alpha for Observer and Perpetrator Justice Sensitivity and Their Bivariate Correlation Across Studies.

JS Scales in each Study	*M*	*SD*	Cronbach’s α	*r* [95% CI]
Study 1				
Observer JS	3.14	1.17	.75	.32*** [.17, .45]
Perpetrator JS	3.69	1.14	.75	
Study 2				
Observer JS	3.06	1.16	.75	.42*** [.30, .52]
Perpetrator JS	3.64	1.15	.71	
Study 3				
Observer JS	3.13	0.84	.87	.55*** [.47, .63]
Perpetrator JS	3.62	0.96	.91	
Study 4				
Observer JS	2.98	0.98	.93	.55*** [.50, .60]
Perpetrator JS	3.53	1.04	.94	
Study 4b				
Observer JS	2.87	0.94	.90	.62*** [.57, .67]
Perpetrator JS	3.52	0.97	.91	
Study 5				
Observer JS	3.22	0.83	.89	.52*** [.46, .57]
Perpetrator JS	3.76	0.86	.91	

*Note.* Response options ranged from 0 (*Not at all)* to 5 (*Absolutely*). *M* = mean; *SD* = standard deviation; *r* = Pearson correlation coefficient; CI = confidence interval; JS = justice sensitivity.

*** *p* < .001.

#### Statistical analyses

We followed a multilevel modeling approach, clustering punishment decisions in each round of the 3PPG (Level 1) within participants (Level 2). The model included the participants’ ID as random factor, ambiguity of the norm violation as Level 1 fixed factor (0 = *No ambiguity*, 1 = *Ambiguity*), Observer JS and Perpetrator JS as Level 2 fixed factors (grand-mean centered), and the respective two cross-level interactions.

## Results and Discussion

Table 2 displays the descriptive statistics of 3PP in each experimental condition.

**Table 2.** Descriptive Statistics of Punishment per Experimental Condition in Studies 1 to 3.

	Punishment		
Experimental Condition	*M*	*SD*	%
Study 1			
No ambiguity	10.83	7.71	84.62
Ambiguity	6.11	6.04	75.23
Study 2			
No ambiguity	9.69	7.74	77.43
Ambiguity	4.99	6.18	65.93
Study 3			
No ambiguity	6.43	7.32	59.46
Ambiguity	3.35	5.02	52.62

*Note.* Punishment = Amount of ECUs (1 ECU = 1 Euro) subtracted from Person A. *M* and *SD* are mean and standard deviation of the sum of ECUs punished across decisions to unequal splits from Person A (i.e., €[0–4] ECUs) to Person B. % = percentage of participants who punished at least 1 ECU. For descriptive statistics split by decision of the strategy method, including those to fair distributions by Person A, see Supplemental Figures S3–S5. ECU = experimental currency unit.

**Figure 1.**
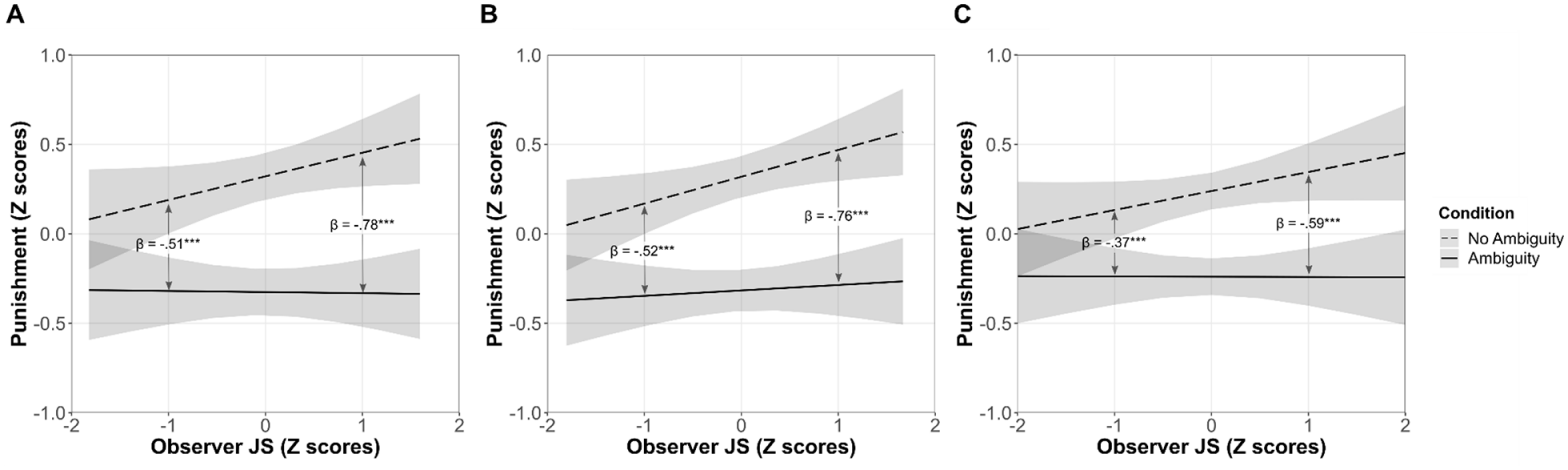
Two-way interaction between ambiguity and observer JS in Studies 1 (A), 2 (B), and 3 (C). *Note.* Standardized regression coefficients represent the effect of ambiguity at 1 SD above and 1 SD below the mean of Observer JS, based on simple slope analyses. Bandwidths indicate 95% CIs. *SD* = standard deviation; JS = justice sensitivity; CI = confidence interval. ***p < .001.

The multilevel models from Studies 1 and 2 (see Table 3) consistently showed that the ambiguity of the norm violation significantly reduced 3PP—supporting H1. Moreover, in both studies, Observer JS and not Perpetrator JS significantly moderated the effect of ambiguity. As Figure 1 summarizes, simple slope analyses showed within-subject differences indicating that participants with high Observer JS (i.e., 1 *SD* above the mean) punished significantly less under ambiguity than under no ambiguity. Participants with low Observer JS (i.e., 1 *SD* below the mean) did also significantly decrease their 3PP under ambiguity; yet, the observed interaction effect indicated that they decreased 3PP to a significantly lesser extent than participants with high Observer JS.

**Table 3.** Tested Multilevel Model on Punishment in Studies 1 to 3.

Parameters	Study 1			Study 2			Study 3		
	β [95% CI]	*t*	*p*	β [95% CI]	*t*	*p*	β [95% CI]	*t*	*p*
Ambiguity of norm violation	−.64 [−.74, −.55]	−13.06	**<.001**	−.63 [−.70, −.56]	−17.38	**<.001**	−.48 [−.54, −.41]	−14.47	**<.001**
Perpetrator JS	.06 [−.07, .20]	0.92	.356	−.03 [−.15, .10]	−0.44	.661	.19 [.06, .31]	2.96	.**003**
Observer JS	.13 [.00, .27]	1.89	.060	.14 [.01, .26]	2.17	.**031**	.11 [−.02, .23]	1.70	.089
Ambiguity × Perpetrator JS	.01 [−.09, .12]	0.26	.795	.05 [−.03, .13]	1.25	.212	−.11 [−.18, −.03]	−2.67	.**008**
Ambiguity × Observer JS	−.13 [−.23, −.03]	−2.57	.**011**	−.12 [−.20, −.04]	−2.95	.**003**	−.11 [−.19, −.03]	−2.73	.**006**
Random effects									
σ^2^		20.99			16.19			12.55	
τ_00 ID_		26.85			32.99			25.39	
ICC _ID_		0.56			0.67			0.67	
N _ID_		163			224			281	
Observations		648			896			1,108	
Marginal/Conditional *R*^2^		.118/.613			.108/.706			.094/.700	

*Note.* CI = confidence interval; JS = justice sensitivity; σ^2^ = residual variance; τ_00 ID_ = variance of the intercept; ICC _ID_ = intraclass correlation coefficient; N_ID_ = total number of individuals.

In bold, those p-values indicating statistical significance (*p* < .05).

We had initially predicted this specific pattern for Perpetrator JS (H2a). However, the findings indicated that, first and foremost, Observer JS captured relevant interindividual differences in the reaction to ambiguity of the norm violation—supporting competing H2b.

Notwithstanding the consistent results from Studies 1 and 2, we decided it was necessary to replicate them while excluding that the effect of ambiguity derived from the fixed order of the rounds in the 3PPG. Given that we introduced ambiguity in the two last rounds, our findings could have resulted from a decay in prosocial behavior across game rounds (e.g., Fehr & Gächter, 2002) or end-of-game effects (Andreoni, 1988). Study 3 addressed these concerns.

## Study 3

Study 3 followed the same within-subject design as Studies 1 and 2, but we introduced a “pseudo-randomization” of the order of the rounds in the 3PPG. We counterbalanced across participants which of the four rounds they played first. After this first round, we randomly presented the three other rounds. A practical matter was behind this pseudo-randomization. In case we had identified order effects, the pseudo-randomization would have allowed us to test our hypotheses using the first round to analyze between-subject differences in 3PP across experimental conditions. We explain any other methodological deviation from Studies 1 and 2 below.

### Method

#### Participants

Based on a priori power simulations, we aimed for 300 participants to ensure 95% power to detect the Ambiguity × Observer JS interaction as observed in Study 1 (for details, see Supplemental Section 5). We recruited 311 participants and excluded data of 22 based on one attention check and on the preregistered criteria used in Studies 1 and 2. The final sample consisted of 284 participants, mainly undergraduate students from diverse disciplines (68% women, age range = 18–73 years, *M* = 23.37 years, *SD* = 6.19 years). Participants received a fixed monetary reward of €2.50 and could additionally earn up to €10 in the 3PPG.

#### Procedure

We conducted the study online. After providing informed consent, participants completed the JS scales (embedded between two filler questionnaires) and the 3PPG. With the exception of the pseudorandomized rounds of the 3PPG, any other procedural detail was identical to Studies 1 and 2.

#### Measures

##### JS

We used the 40-item version of the JS Inventory (Schmitt et al., 2010). Ten items served for measuring each JS perspective, including the same two items of the short version.

#### Statistical analyses

We tested the same model as in Studies 1 and 2. Next, we created two factors for examining order effects and introduced them as covariates into our model. At Level 1, the factor *Position* captured the position at which a particular round of the game was presented to a participant (i.e., 0 = *Round 1*, 1 = *Round 2*, 2 = *Round 3*, 3 = *Round 4*). This factor accounted for potential linear effects on 3PP over time. Moreover, we wanted to pay special attention to the effects of the order of presentation of the *ambiguity* conditions. Thus, at Level 2, the factor *Ambiguity Order* captured the six possible randomized orders in which the *ambiguity* (yes) and *no ambiguity* (no) conditions were presented to participants (i.e., 0 = *no, no, yes, yes*; 1 = *yes, yes, no, no*; 2 = *no, yes, no yes*; 3 = *yes, no, yes, no*; 4 = *yes, no, no, yes*; 5 = *no, yes, yes, no*). We tested the main effects of these two factors on 3PP and, more importantly, whether Ambiguity Order moderated the effect of ambiguity or the cross-level interactions with Observer and Perpetrator JS.

### Results and Discussion

As in Studies 1 and 2, ambiguity significantly reduced 3PP, and Observer JS significantly moderated this effect (see Table 3). Simple slope analyses indicated that the effect of ambiguity was more pronounced among those with high (vs. low) Observer JS (see Figure 1). In contrast to Studies 1 and 2, Perpetrator JS significantly moderated the effect of ambiguity. Ambiguity decreased 3PP more pronouncedly among those with high Perpetrator JS, β = −.52, *t*(829) = −11.29, *p* < .001, 95% confidence interval (CI) = [−.61, −.43], than among those with low Perpetrator JS, β = −.33, *t*(826) = −7.36, *p* < .001, 95% CI = [−.42, −.24].

Entering the factors Position and Ambiguity Order as covariates did not significantly increase the model fit, ∆*AIC* = 20.1, χ^2^(31) = 41.85, *p* = .092. As the number of model parameters increased to 36, we simplified the results by presenting the omnibus tests of the main and interaction effects in an analysis of variance (ANOVA) table (Table 4). The main effects of Position and Ambiguity Order were not significant. More importantly, Ambiguity Order did not significantly moderate any main effect or interaction. Note, however, that the Ambiguity × Observer JS remained significant in this model, while the Ambiguity × Perpetrator JS interaction was not significant. Given that both interactions had similar effect sizes in our first model, we do not attribute this null effect to an issue of statistical power. Instead, we believe that the Ambiguity × Perpetrator JS interaction was simply spurious because it was sensitive to covariates that were statistically irrelevant.

**Table 4.** ANOVA Table of Multilevel Model Accounting for Order Effects in Study 3.

Parameters	Punishment			
	*df*	***F***	***p***	$\eta_{p}^{2}$
Ambiguity of norm violation	1, 812	205.02	**<.001**	.197
Perpetrator JS	1, 264	3.55	.061	.004
Observer JS	1, 264	0.57	.452	.001
Position	1, 811	0.93	.336	.001
Ambiguity Order	5, 264	1.76	.122	.010
Ambiguity × Perpetrator JS	1, 811	3.08	.080	.004
Ambiguity × Observer JS	1, 812	7.39	.**007**	.009
Ambiguity × Ambiguity Order	5, 812	2.03	.072	.012
Perpetrator JS × Ambiguity Order	5, 264	0.78	.567	.005
Observer JS × Ambiguity Order	5, 264	0.70	.623	.004
Ambiguity × Perpetrator JS × Ambiguity Order	5, 811	1.68	.136	.010
Ambiguity × Observer JS × Ambiguity Order	5, 812	0.32	.904	.002

*Note. df* = numerator and denominator degrees of freedom calculated with Satterthwaite’s method. ANOVA = analysis of variance; JS = justice sensitivity.

In bold, those p-values indicating statistical significance (*p* < .05).

Study 3 closely replicated the findings from Studies 1 and 2, while the experimental setup served to unconfound ambiguity effects from potential order effects. Thus, Studies 1 to 3 provided consistent evidence for a decrease of 3PP under ambiguity (H1), especially among participants with high Observer JS. The three studies showed that Observer JS positively predicted the 3PP of clear norm violations, while Observer JS did not predict 3PP when the norm violation was ambiguous. Put differently, when the norm violation was clear, people with high (vs. low) Observer JS showed higher 3PP; however, under ambiguity, they pronouncedly reduced their 3PP and, consequently, we observed people with high and low Observer JS exerting similarly low 3PP. Previous research suggests that individuals with high other-oriented JS (e.g., Observer and Perpetrator JS) are less driven by selfish temptations (e.g., Baumert, Schlösser, & Schmitt, 2014; Lotz et al., 2013). Hence, it seemed implausible that individuals with high Observer JS reduced 3PP because ambiguity offered them moral wiggle room to avoid incurring costs. Instead, it is more conceivable that they hesitated to punish because ambiguity introduced risk of becoming unfair themselves (i.e., type I error concerns).

In sum, the examination of the moderating role of JS was informative, highlighting the importance of justice concerns for understanding the decrease of 3PP under ambiguity. However, it only offered indirect evidence regarding the motivational underpinnings of the effect of ambiguity. Therefore, in Studies 4 and 4b, we used an alternative experimental approach to fill this gap by investigating whether and why third parties would resolve the ambiguity.

## Study 4

In Study 4, we introduced a third condition to our experimental design, where third parties had the opportunity to resolve the ambiguity by revealing perfect information about the norm violation before their punishment decision. Giving third parties the chance to resolve the ambiguity should help distinguish more clearly the aforementioned motivational mechanisms underlying the effect of ambiguity. Specifically, we argued that those who were motivated to react against unfairness in principle but hesitated to punish under ambiguity due to the risk of punishing unfairly, would take any chance to resolve the ambiguity to alleviate their type I error concerns. Conversely, those who aimed to remain passive to avoid incurring costs could keep and capitalize on the ambiguity as a situational justification to passiveness. Hence, the new condition allowed us to examine (a) whether those third parties who opted for resolving the ambiguity—arguably alleviating any type I error concern—punished the disambiguated norm violation, and (b) whether those third parties who opted for keeping the situation ambiguous—and thus, a moral wiggle room to avoid incurring costs—remained passive.

We preregistered (https://osf.io/ym4r3) that, in the new condition, Observer and Perpetrator JS would positively predict whether third parties resolved the ambiguity (H3a–b) because we argued that both dispositional measures might capture type I error concerns. Moreover, we predicted that those third parties who resolve the ambiguity would show higher 3PP than those who did not resolve the ambiguity (H4). Under the assumption that those who resolved the ambiguity were genuinely motivated to disambiguate the situation to address any potential norm violation, we further hypothesized that, once they had resolved the ambiguity, they would show even higher 3PP than those in the *no ambiguity* condition (H5). In contrast, we assumed that those who did not resolve the ambiguity aimed to avoid costs and exploit the ambiguity as moral wiggle room; therefore, we predicted that they would show the lowest 3PP, even relative to those in the *ambiguity* condition (H6).

Moreover, we administered a postexperimental questionnaire to gauge different considerations that third parties might have when deciding (a) whether to resolve the ambiguity and (b) whether to punish, including the discussed type I error concerns and cost avoidance.

### Method

#### Design

Study 4 followed a between-subject design, with three experimental conditions: *no ambiguity, ambiguity*, and the additional condition where participants faced the same ambiguous norm violation, but, before their punishment decision, they could *resolve/not resolve* the ambiguity by incurring a minor cost. We decided to make the option to resolve the ambiguity costly to distinguish clearly third parties who would resolve the ambiguity to inform their punishment decision to avoid punishing unfairly from those who would resolve the ambiguity merely out of curiosity. We based this decision on the results of Study 4b, a prior study similar to Study 4, which only differed in that resolving the ambiguity was cost-free. In Study 4b, people who resolved the ambiguity reported that curiosity partly drove their decision. For a matter of conciseness, we report the results of Study 4b in the Supplemental Section 4.

Different from the strategy method used in Studies 1 to 3, in Studies 4 and 4b, participants only decided in the role of Person C and reacted to a specific distribution by Person A (i.e., a distribution of 1 ECU, given an endowment of 10 ECUs; for details about how we elicited this distribution from Person A, see Supplemental Section 6). The cost of punishment and compensation was fixed (i.e., for every 1 ECU punished or compensated, a cost of 0.5 ECU).

#### Participants

A priori power analyses indicated that a sample size of *N* = 300 would suffice to detect an interaction effect as observed in Study 3 with 80% statistical power (for details, see Supplemental Section 5). Because we aimed to compare the subsets of participants who decided (not) to resolve the ambiguity in our third experimental condition with the *no ambiguity* and *ambiguity* conditions, we planned to collect double the sample size (*N* = 600).

We recruited 857 participants from a German online panel. We excluded data from 52 who did not finish the study and 119 who failed preregistered comprehension checks about the 3PPG and the manipulation of ambiguity (e.g., “How much [ECUs] does Person A receive?”). The resulting sample was 686 participants, overall with high education (62%) and a more representative demographical distribution than our previous studies (50% women, age range = 18–82 years, *M* = 46.53 years, *SD* = 14.41 years). Participants received a fixed monetary reward of €2.00, and they could earn up to €5.00 in the 3PPG.^2^

#### Procedure

After providing informed consent and demographic information, participants answered the JS scales, embedded in between two filler questionnaires. Next, they were randomly assigned to one of the three conditions and played the 3PPG as Person C.

The *no ambiguity* and *ambiguity* conditions were identical to those in Studies 1 to 3. In the *resolve/not resolve* condition, participants received the same information as those in the *ambiguity* condition, with the difference that, before the punishment and compensation decisions, we asked them whether they would like to know how many points Person A had originally received. If they answered “yes,” we disclosed that Person A had received an endowment of 10 ECUs; if they answered “no,” this information remained unknown. As discussed above, we made this decision costly. To do so without altering the payoff structure of the 3PPG, all participants in every experimental condition could win a €5.00 voucher in a raffle with 1/20 probability at the end of the study. In the *resolve/not resolve* condition, the probability of winning the raffle was conditional on the decision to resolve the ambiguity. Specifically, if participants decided to resolve the ambiguity, the probability of earning the voucher would decrease to 1/30.

Participants then made their punishment and compensation decisions. Finally, they completed the postexperimental questionnaire.

#### Measures

##### Decision to resolve ambiguity

We coded the dichotomous decision as 0 = *No*, 1 = *Yes*.

##### 3PP

The measure of 3PP was the total amount of ECUs that participants wished to subtract from Person A (0–10 ECUs).

##### JS

We used the 40-item JS Inventory.

#### Postexperimental questionnaire

The postexperimental questionnaire included ad hoc-generated items to capture potential considerations regarding the decisions of whether or not (a) resolving the ambiguity, and (b) punishing Person A. Participants in the *resolve/not resolve* condition reported their agreement with items capturing considerations underlying their decision to *resolve the ambiguity*, using a 6-point Likert-type scale (0 = *Not at all*, 5 = *Absolutely*). Participants in all conditions similarly reported their agreement with items assessing considerations underlying their decision to *punish Person A*.

Here, we only report the results from items intended to capture the theorized mechanisms of type I error concerns and cost avoidance (for the complete questionnaire, see Supplemental Table S13). Table 5 summarizes these items and their respective factor loadings according to a *principal component analysis* (PCA). We computed aggregated scores for the pairs of items that loaded onto the same factor.

**Table 5.** Selected Items From Postexperimental Questionnaire and Factor Loadings Based on Principal Component Analysis With Oblimin Rotation.

Concerns related to resolving ambiguity	Items		
Unfair decision	1. I’ve been thinking about the risk of being unfair to Person A.		
Cost avoidance	4. My priority was to avoid costs.		
Concerns related to punishment	Items	PC1	PC2
Type I error	1. I was concerned that my decision about Person A could be unfair.	.**87**	.11
	2. I wanted to avoid being unfair toward Person A.	.**84**	−.12
Cost avoidance	17. My priority was to avoid costs.	−.17	.**86**
	18. I have barely taken into account the cost of my decision. **(R)**	−.24	.**79**

*Note.* In bold, those loadings greater than .30. PC = principal component.

#### Statistical analyses

First, we used the data from the *no ambiguity* and *ambiguity* conditions in a multiple regression model to test whether our previous findings replicated. The model regressed 3PP on ambiguity, Observer JS, Perpetrator JS, and the Ambiguity × Observer JS and Ambiguity × Perpetrator JS interactions.

Second, with the data from the *resolve/not resolve* condition, we used logistic regression to test whether Observer and Perpetrator JS predicted the decision to resolve the ambiguity.

Finally, to test for the expected differences in 3PP across conditions (*ambiguity* and *no ambiguity*) and/or self-selected groups (*resolved* and *not resolved*), we fitted three independent regression models with different data subsets. Each model included one dummy-coded variable as predictor, which captured the two groups that the model aimed to compare: Dummy 1 (0 = *Not resolved*, 1 = *Resolved*), Dummy 2 (0 = *No ambiguity*, 1 = *Resolved*), and Dummy 3 (0 = *Ambiguity*, 1 = *Not resolved*), respectively.

### Main Results

**Figure 2.**
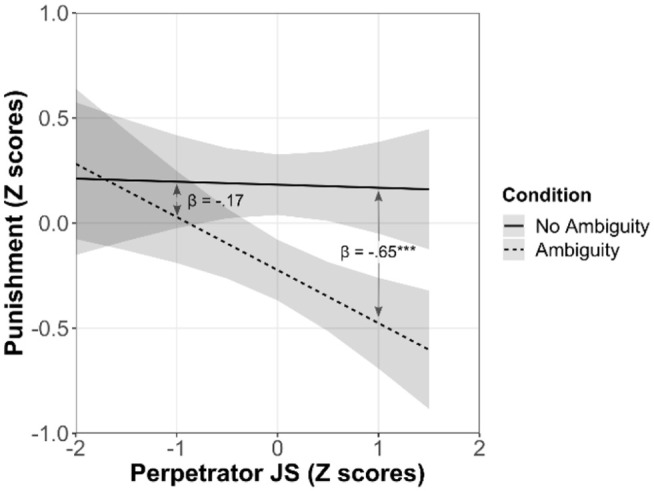
Two-way interaction between ambiguity and Perpetrator JS in Study 4. *Note.* Standardized regression coefficients represent the effect of ambiguity at above and 1 SD below the mean of Perpetrator JS, based on simple slope analyses. Bandwidths indicate 95% CIs. *SD* = standard deviation; JS = justice sensitivity; CI = confidence interval. ***p < .001.

We found that 3PP was significantly lower in the *ambiguity* condition than in the *no ambiguity* condition—supporting H1 (see Table 6). Different from previous studies, the Ambiguity × Observer JS interaction was not significant—not supporting H2b. Instead, we observed a significant Ambiguity × Perpetrator JS interaction. Simple slopes indicated that the effect of ambiguity was only significant among those with high Perpetrator JS (see Figure 2).

**Table 6.** Tested Multiple Regression Model on Punishment in Study 4.

Parameters	β [95% CI]	*t*	*p*
Ambiguity of norm violation	−.41 [−.62, −.21]	−3.94	**<.001**
Perpetrator JS	−.01 [−.19, .16]	−0.17	.865
Observer JS	.03 [−.14, .20]	0.34	.737
Ambiguity × Perpetrator JS	−.24 [−.48, −.01]	−2.01	.**045**
Ambiguity × Observer JS	.03 [−.21, .27]	0.27	.788
Observations	345		
*R*^2^/Adj. *R*^2^	.070/.056		

*Note.* CI = confidence interval; JS = justice sensitivity.

In bold, those p-values indicating statistical significance (*p* < .05).

In the *resolve/not resolve* condition, a third of the participants resolved the ambiguity despite the incurred cost (36.07%), whereas most participants decided not to resolve the ambiguity (63.93%). The logistic regression model showed that this decision was not predicted by Observer JS, Wald(1) = 0.61, *p* = .539, odds ratio (OR) = 1.09, 95% CI = [0.82, 1.46], nor Perpetrator JS, Wald(1) = 0.77, *p* = .439, OR = 1.12, 95% CI = [0.85, 1.47]—not supporting H3a–b.

**Figure 3.**
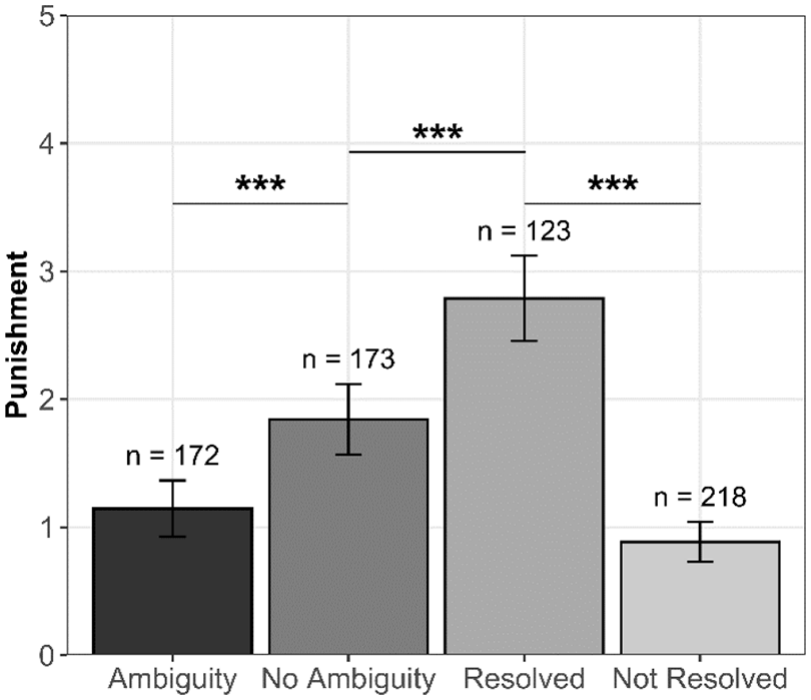
Levels of 3PP in experimental conditions and self-selected groups in Study 4. *Note.* Error bars indicate 95% CIs. CI = confidence interval. ***p < .001.

Next, we compared the levels of 3PP across the different conditions/groups of participants (see Figure 3). The first regression model showed that those who resolved the ambiguity punished significantly more than those who did not—supporting H4; Dummy 1, β = 1.11, *t*(339) = 11.58, *p* < .001, 95% CI = [0.92, 1.29]. The second model indicated that those who resolved the ambiguity also punished significantly more than those in the *no ambiguity* condition—supporting H5; Dummy 2, β = .50, *t*(294) = 4.34, *p* < .001, 95% CI = [.27, .72]. The third model showed that the level of 3PP did not significantly differ between those who did not resolve the ambiguity and those in the *ambiguity* condition—not supporting H6; Dummy 3, β = −.20, *t*(388) = −1.95, *p* = .052, 95% CI = [−.40, .00].

### Exploratory Results

**Figure 4.**
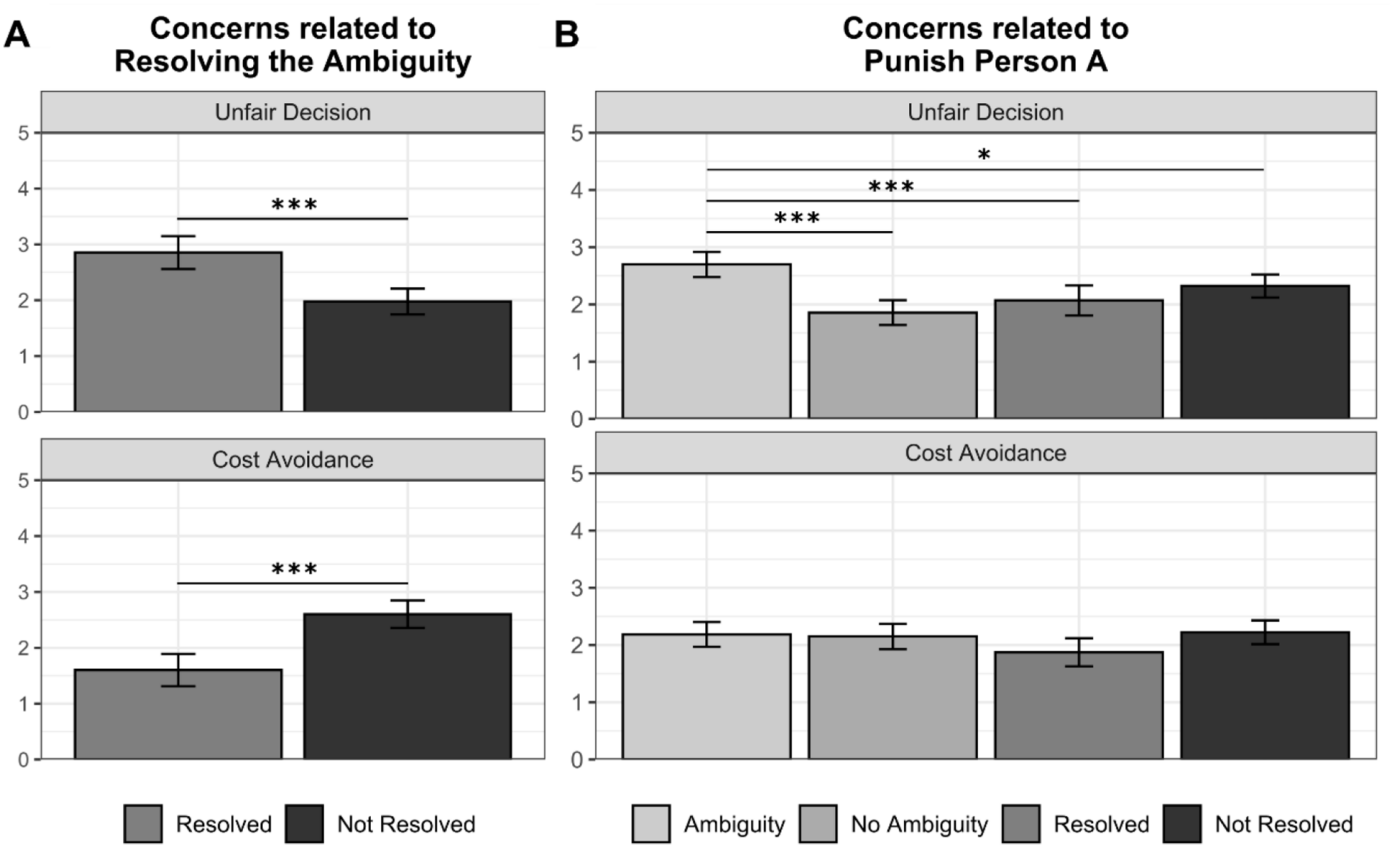
Concerns associated with the decision to resolve the ambiguity (A) and the decision to punish (B) in Study 4. *Note. p*-values correspond to Welch independent sample *t* tests (A) and linear regressions including the experimental group as predictor (B). Error bars indicate 95% CIs. CI = confidence interval. *p < .05. ***p < .001.

With the postexperimental questionnaire, we first aimed to elucidate which considerations drove participants to resolve the ambiguity. For this purpose, we analyzed whether the considerations that participants reported regarding their decision of resolving the ambiguity differed between those who resolved the ambiguity and those who did not. Indeed, the former reported significantly higher concerns about making an unfair decision and significantly lower concerns about avoiding costs (see Figure 4A).

Next, we intended to clarify if ambiguity affected considerations relating to the participants’ decision to punish Person A. Thus, we examined whether the considerations that participants reported differed across the different experimental groups, taking the ambiguity condition as reference (see Figure 4B). We observed that participants in the *ambiguity* condition reported significantly higher concerns about making an unfair decision than those in the *no ambiguity* condition and those who resolved or kept the ambiguity in the *resolve/not resolve* condition. We did not observe significant differences in cost avoidance.

**Figure 5.**
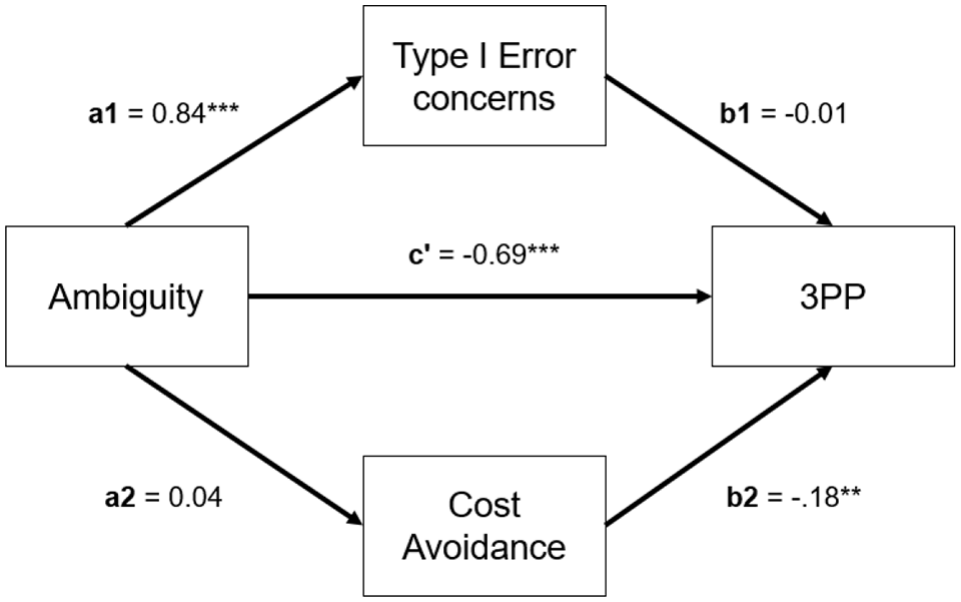
Exploratory parallel mediational model tested in Study 4. **p < .01. ***p < .001.

Finally, we explored whether type I error concerns and cost avoidance mediated the effect of ambiguity on 3PP (see Figure 5). For this purpose, we only used the data from the ambiguity and the no ambiguity conditions. The mediation model showed that ambiguity was positively associated with type I error concerns but not cost avoidance, whereas only cost avoidance negatively predicted 3PP. Both indirect effects were not significant, *a*_1_*b*_1_ = −0.01, 95% CI = [−.11, .10]; *a*_2_*b*_2_ = −0.01, 95% CI = [−.07, .05] (bootstrap 5,000 iterations).

### Discussion

We again replicated the effect of ambiguity and, in contrast to Studies 1 to 3, we found Perpetrator and not Observer JS to moderate this effect. Moreover, Study 4 provided mixed evidence regarding the underlying mechanisms of the effect of ambiguity.

On one hand, Study 4 teased apart people who were susceptible to ambiguity out of different motivations.

First, those participants who resolved the ambiguity were motivated to punish the disambiguated norm violation, as their higher 3PP indicate. We infer from the lower 3PP in the *ambiguity* (vs. *no ambiguity*) condition that these participants would have refrained from punishing if they had not had the option to resolve the ambiguity (and, thus, to know whether their punishment was unfair). In fact, they willingly incurred additional costs for revealing that information, which highlights that they cared about addressing correctly a norm violation while avoiding being unfair themselves. The postexperimental questionnaire partly supported this notion by showing that these participants reported higher concerns about making an unfair decision than those who did not resolve the ambiguity.

Second, those people who opted for not resolving the ambiguity leant toward cost avoidance. They chose the option that did not entail costs, although the costs were negligible (i.e., between €5.00 with probability 1/20 and 1/30, the expected value difference is €0.08). Moreover, they exerted low 3PP, similarly to those in the *ambiguity* condition, but significantly less than those participants who resolved the ambiguity and those in the *no ambiguity* condition. These findings could tentatively align with the literature on moral wiggle room, as they show the inclination of some individuals toward the avoidance of individual costs under situational ambiguity.

On the other hand, part of our findings was not consistent with the theorized mechanisms. Unexpectedly, we did not find Observer and Perpetrator JS to predict the decision to resolve the ambiguity, although both JS scales correlated significantly positively with concerns about punishing unfairly (see Supplemental Table S14). Furthermore, we did not observe concerns about punishing unfairly and cost avoidance to mediate the effect of ambiguity on 3PP. Under ambiguity, participants reported higher concerns about punishing unfairly, but these did not decrease 3PP.

In Study 4b, where resolving the ambiguity was not costly, offered comparable results regarding the *resolve/not resolve* condition. However, we found the Ambiguity × Observer JS to be significant and type I error concerns to mediate the effect of ambiguity (see Supplemental Section 4). We will address and frame these inconsistencies in the section “General Discussion.”

## Study 5

We designed Study 5 to exclude a potential threat to the internal validity of our ambiguity manipulation. As a reminder, in our *no ambiguity* condition, we fixed Person A’s endowment to 10 ECUs, whereas in the *ambiguity* condition Person A’s endowment randomly varied between 2 and 10 ECUs. In the latter condition, third parties could make assumptions about the endowment’s probability distribution (e.g., uniform distribution^3^) and consequently infer a lower expected value of the endowment in the *ambiguity* condition than in the *no ambiguity* condition. If this was the case, the lower expected value in the *ambiguity* condition could explain the reduction of 3PP.

For ruling out this explanation, in Study 5, we separately manipulated *ambiguity* (no ambiguity vs. ambiguity) and Person A’s endowment *expected value* (low vs. high). Specifically, in the *no ambiguity*/*low expected value* condition, Person A received a fixed endowment of 6 ECUs, which corresponded to the expected value of our original *ambiguity* condition, now relabeled *ambiguity/low expected value* condition (i.e., random endowment from 2 to 10 ECUs). In the *ambiguity/high expected value* condition, Person A received a random endowment from 2 to 18 ECUs, the expected value of which corresponded to the fixed endowment of our original *no ambiguity* condition, now relabeled *no ambiguity/high expected value* condition (i.e., endowment of 10 ECUs). Hence, our first preregistered hypothesis (https://osf.io/s7rza) was that the ambiguity effect would remain significant across expected values (H7), even if the interaction between ambiguity and expected value turned out significant.

In Study 5, we continued exploring the underlying mechanisms of the effect of ambiguity. For this purpose, we measured the third parties’ type I error concerns through a postexperimental questionnaire as in Study 4. We predicted that the introduction of ambiguity would relate to these type I error concerns and that the latter would predict 3PP (H8). Moreover, we included Social Value Orientation (SVO; van Lange et al., 1997), as an additional validated measure of fairness concerns, to explore whether social preferences, and specifically, inequality aversion, played a similar role under ambiguity to the one JS arguably played (for results about this question, see Supplemental Section 8).

### Method

#### Design

We used a 2 × 2 between-subject design, manipulating *ambiguity* (no ambiguity vs. ambiguity) and the endowment’s *expected value* (low vs. high; see Table 7). As in Study 4, participants made a single decision in response to a specific unfair distribution by Person A (i.e., a distribution of 1 ECU, given an endowment of 10 ECUs). The cost of punishment and compensation was 0.5 ECU per every ECU that they punished or compensated.

**Table 7.** Between-Subject Design of Study 5.

Expected Value × Ambiguity	No ambiguity	Ambiguity
Low expected value	6 ECUs	[2 ECUs–10 ECUs]
High expected value	10 ECUs	[2 ECUs–18 ECUs]

*Note.* The shaded cells correspond to the original conditions compared in Studies 1 to 4. ECUs = experimental currency units.

#### Participants

Under the possibility of observing a smaller ambiguity effect in the *low expected value* conditions, we conducted a safeguard power analysis. We considered the smallest ambiguity effect from our previous studies (i.e., *d* = −.38 in Study 4) and took as a reference the lower bound of its 90% confidence interval (i.e., *d* = −.21). To detect this effect size in a one-tailed *t* test (α = .05), we would need 780 observations to guarantee 90% statistical power.

The present study consisted of two sessions (see justification below). To counter the dropout rates between sessions and the exclusion based on preregistered criteria, we over-recruited 1,116 participants from a German online panel. Of these, 829 completed both sessions (i.e., 26% dropout rate). We excluded data of 51, who failed preregistered comprehension checks about the 3PPG. Therefore, the resulting sample consisted of 778 participants, mostly undergraduate students from diverse disciplines (77% women, age range = 18–65 years, *M* = 24.04 years, *SD* = 4.75 years). They received a fixed monetary reward of €0.50 per session. In addition, they could earn up to €1.00 in the SVO task and up to €5.00 in the 3PPG, but only if they completed both sessions.

### Procedure

Participants completed two sessions to avoid carry-over effects between the payoffs of the SVO task and the 3PPG.

In Session 1, participants completed the JS and SVO measures and provided demographic information. We contacted participants 12 hr later to take part in Session 2, where we randomly assigned participants to play the 3PPG in the role of Person C in one of the experimental conditions. They subsequently completed the postexperimental questionnaire.

#### Measures

##### 3PP

As in Study 4, we assessed 3PP as the total amount of ECUs that participants wished to subtract from Person A (0–10 ECUs).

##### JS

We used the 40-item JS Inventory.

#### Postexperimental questionnaire

##### Type I error concerns

Participants reported their agreement with the following item: “I was concerned that my decision about Person A could be unfair” (0 = *Not at all*, 5 = *Extremely*). Note that we deviated from our preregistered measure by dropping those items that did not capture concerns about making an unfair decision explicitly (e.g., “I was concerned about feeling like a malefactor”), as these could compromise the construct validity of our measure (for analyses with all preregistered items, see Supplemental Section 8).

In addition, the questionnaire measured participants’ perceptions about the situation that we used as manipulation checks.

##### Perceived unfairness of Person A’s decision

We used two unipolar scales to measure how *fair* and how *unfair* participants found Person A’s distribution (0 = *Not at all*, 5 = *Extremely*). We computed a difference score, with higher scores representing higher unfairness.

##### Perceived ambiguity of the norm violation

We used two items to measure the perceived ambiguity when judging Person A’s decision: “How uncertain do you feel regarding your evaluation of Person A’s decision?” and “How difficult do you find to judge Person A’s decision?” (Spearman–Brown’s estimate, ρ = .83).

#### Statistical analyses

We first examined the manipulations checks through two independent regression models. We, respectively, regressed perceived unfairness and perceived ambiguity of the norm violation on ambiguity (0 = *No ambiguity*, 1 = *Ambiguity*), expected value (0 = *Low expected value*, 1 = *High expected value*), and their interaction.

Next, we tested H7 by regressing 3PP on ambiguity, expected value, and their interaction.

With regard to H8, we tested a mediation model, which included ambiguity as predictor, type I error concerns as mediator, and 3PP as criterion.

### Results

#### Manipulation checks

**Figure 6.**
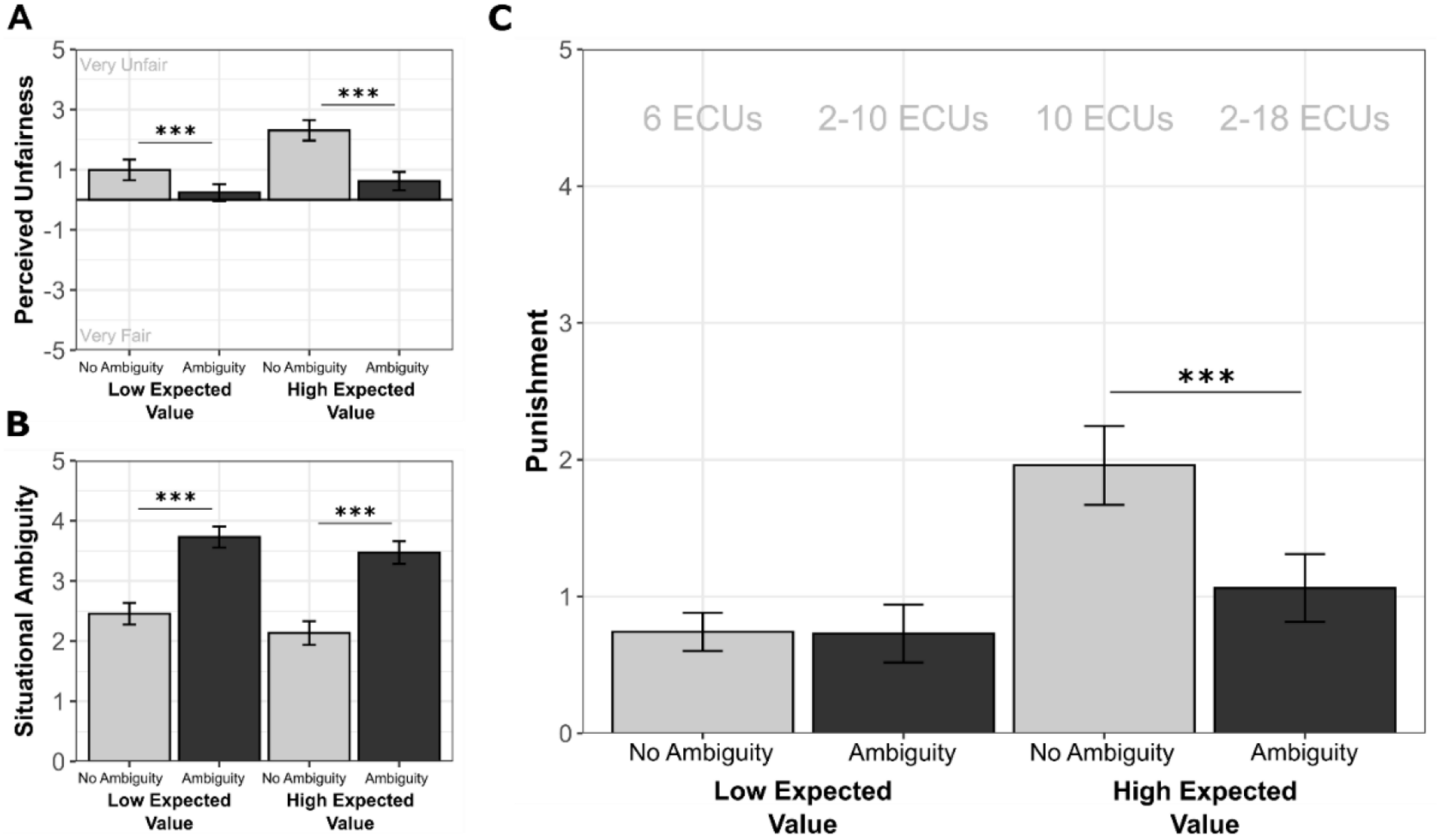
Levels of perceived unfairness (A), perceived situational ambiguity (B), and 3PP (C) across experimental conditions in Study 5. *Note*. Error bars indicate 95% CIs. . CI = confidence interval. ***p < .001.

For participants’ perceived unfairness (Figure 6A), we found a significant Ambiguity × Expected Value interaction term, β = −.39, *t*(768) = −2.88, *p* = .004, 95% CI = [−.66, −.13]. Participants perceived significantly less unfairness under *ambiguity* than under *no ambiguity* in the *low expected value* conditions—as the significant conditional effect of Ambiguity indicated, β = −.32, *t*(768) = −3.31, *p* < .001, 95% CI = [−.51, −.13]—and this difference was significantly more pronounced in the *high expected value* conditions. At the same time, participants perceived significantly more unfairness when Person A had 10 ECUs (*high expected value*) than when Person A had 6 ECUs (*low expected value*) in the *no ambiguity* conditions—as the significant conditional effect of Expected Value indicated, β = .55, *t*(768) = 5.76, *p* < .001, 95% CI = [.37, .74]. Yet, this difference was significantly less pronounced in the *ambiguity* conditions.

Regarding perceived ambiguity (Figure 6B), we did not find a significant Ambiguity × Expected Value interaction term. Participants perceived significantly higher ambiguity in the *ambiguity* than in the *no ambiguity* conditions—as the Ambiguity term indicated, β = .87, *t*(769) = 9.66, *p* < .001, 95% CI = [0.70, 1.05]—and significantly lower ambiguity in the *high* (vs. *low*) *expected value* condition—as the Expected Value term indicated, β = −.22, *t*(769) = −2.43, *p* = .015, 95% CI = [−.40, −.04].

#### Main analyses

The model for 3PP (Figure 6C) revealed a significant Ambiguity × Expected Value interaction term, β = −.52, *t*(774) = −3.81, *p* < .001, 95% CI = [−.79, −.25]. Participants punished significantly less under *ambiguity* than under *no ambiguity* in the *high expected value* conditions. This was not the case in the *low expected value* conditions—as the nonsignificant Ambiguity term indicated, β = −.01, *t*(774) = −0.069, *p* = .945, 95% CI = [−.20, .18]. Furthermore, participants punished significantly more in the *high* (vs. *low*) *expected value* condition under *no ambiguity*—as the significant Expected Value term indicated, β = .72, *t*(774) = 7.40, *p* < .001, 95% CI = [.53, .91]—but this difference was less pronounced under *ambiguity*.

**Figure 7.**
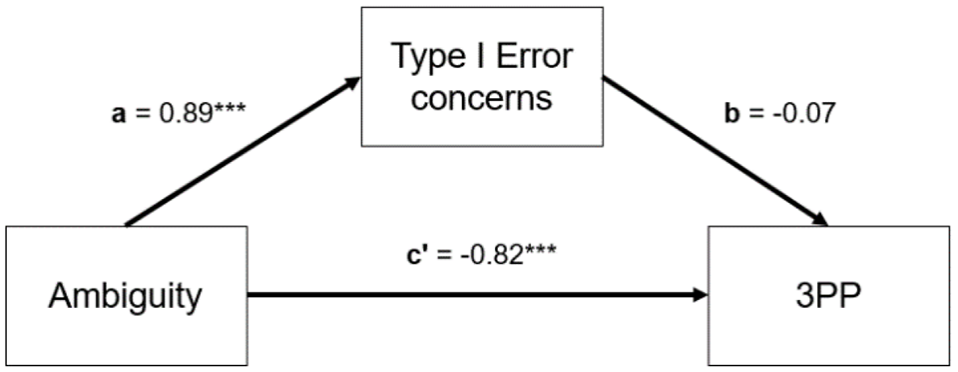
Preregistered mediation model tested in Study 5. ***p < .001.

Because we only observed the ambiguity effect in the *high expected value* conditions, we used this data subset to test the mediation model. The model showed that ambiguity significantly predicted type I error concerns and 3PP; however, type I error concerns did not predict 3PP (Figure 7). The indirect effect of ambiguity on 3PP through type I error concerns was not significant, *ab =* −0.06, 95% CI = [−.16, .03] (bootstrap 5,000 iterations).

### Exploratory Results

**Figure 8.**
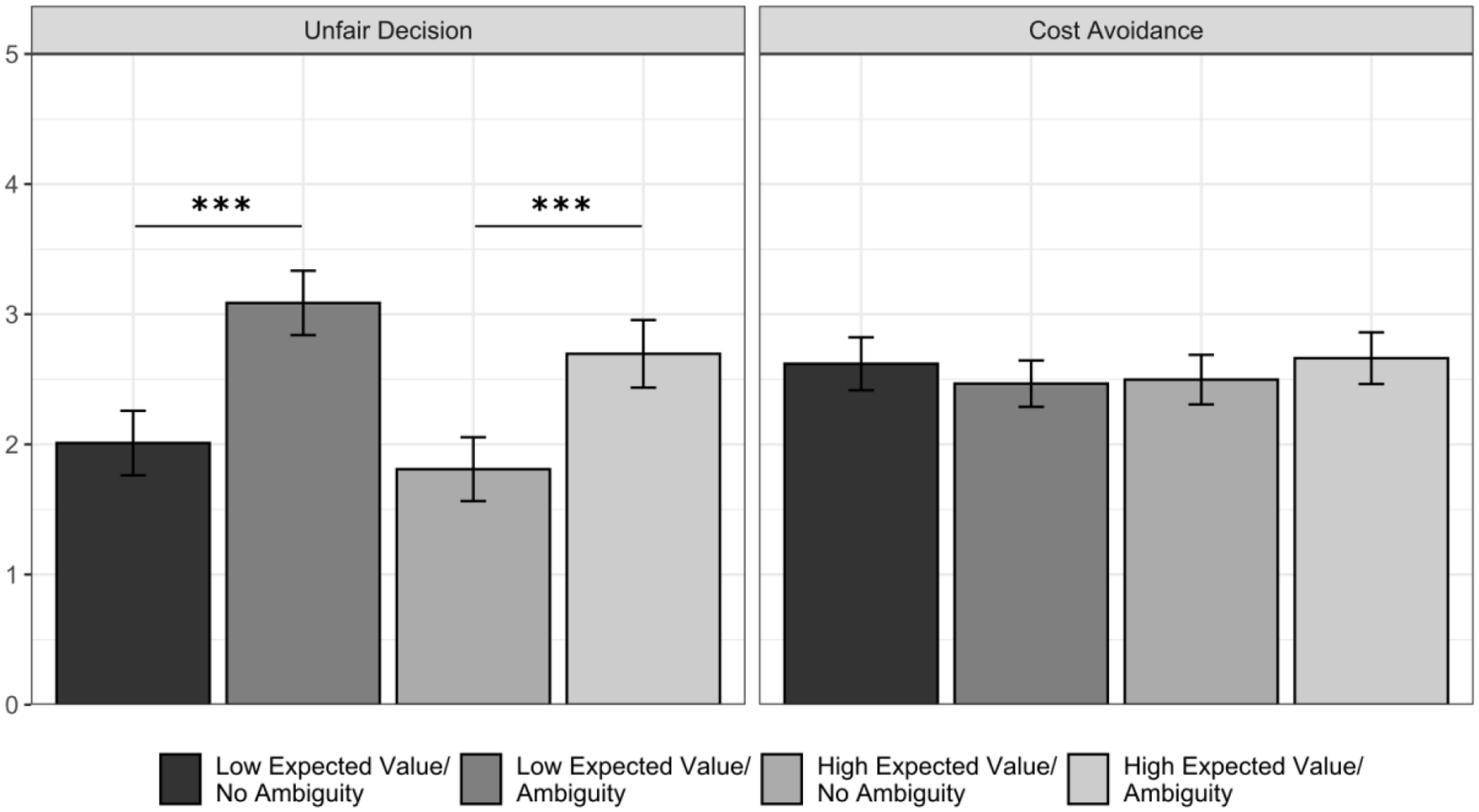
Differences in types of concerns associated with the decision to punish in Study 5. *Note. p*-values correspond to pairwise comparisons across ambiguity and expected value with Bonferroni correction. Error bars indicate 95% CIs. CI = confidence interval. ***p < .001.

We summarized the secondary findings from the postexperimental questionnaire in Figure 8. Because they offered a similar picture to Study 4, we will not discuss them further.

Finally, our secondary results about the role of JS showed that Observer JS moderated neither the effect of ambiguity (even when only considering the *high expected value* conditions) nor the effect of expected value (see Supplemental Table S17).

### Discussion

Study 5 established that ambiguity of the norm violation reduced 3PP when holding constant the expected value of Person A’s endowment, excluding the discussed threat to the internal validity of our ambiguity manipulation. Further aspects of our results need special attention.

The ambiguity effect emerged in the *high expected value* conditions, but not in the *low expected value* conditions. The pattern of results indicates that this was the case because 3PP was unexpectedly low in the *no ambiguity*/*low expected value* condition. In light of the low perceived unfairness in the *low expected value* conditions, it seems plausible that most participants did not perceive the behavior of Person A as norm violation, even without ambiguity. By contrast, in the *high expected value* condition, people perceived higher unfairness under *no ambiguity* than under *ambiguity*, which could explain why the ambiguity effect held. These results suggest that ambiguity might decrease 3PP partly because people might be less likely to perceive the norm violation. This could have been an alternative explanation for the Ambiguity × Observer JS interaction observed in previous studies: people high in Observer JS could have punished less under *ambiguity* because they did not perceive unfairness given the lower expected value. Yet, Study 5 refuted this notion by showing that Observer JS (and the SVO index of inequality aversion; see Supplemental Tables S17–S18) did not moderate the effect of expected value.

Beyond the aforementioned differences in perceived unfairness, the ambiguity effect also related to how difficult people found to judge whether Person A behaved unfairly or not, as the levels of perceived ambiguity indicated. This was apparent in both the *high expected value* and *low expected value* conditions, with participants reporting to struggle more to judge Person A’s behavior under *ambiguity* (vs. *no ambiguity*). If the ambiguity effect on 3PP only emerged in the *high expected value* conditions, it could be because, under *no ambiguity*, participants reported not to struggle when judging Person A’s behavior as clearly unfair, and accordingly, showed higher 3PP. In contrast, in the *no ambiguity/low expected value* condition, participants reported not to struggle but perceived less unfairness.

Taken together, our findings suggest that, under ambiguity, third parties are less likely to perceive norm violations because ambiguity hinders this perception, and therefore, 3PP is expectedly lower.

Finally, we observed higher type I error concerns under ambiguity. Yet, we did not find these to mediate the effect of ambiguity on 3PP. We will delve into this unexpected result when framing it together with the results of Studies 4 and 4b in the section “General Discussion”.

## General Discussion

Despite the crucial role of 3PP for the maintenance of social norms (Fehr & Fischbacher, 2004), this behavior might be less prevalent than previous lab studies suggest, as some have discussed (Pedersen et al., 2018). In these studies, a norm violation was generally easily identifiable. By contrast, in real-life settings, third parties often receive ambiguous information, rendering the interpretation of a situation as a norm violation complicated. Our research highlights that the ambiguity of the norm violation can indeed constitute a critical boundary of 3PP.

In six studies, we consistently observed that, under ambiguity (i.e., if third parties received imperfect information affecting the identification of the norm violation), 3PP decreased, compared with when perfect information was provided. This effect of ambiguity has theoretical implications for understanding the decision-making preceding 3PP. Explanatory frameworks of bystander intervention have proposed that a critical step for third parties to react against norm violations is to interpret them as such (e.g., Baumert et al., 2013). If the situation entails ambiguity concerning the norm violation, we argued that its interpretation is hampered, which exerts downstream effects on how people ponder over exerting 3PP. Specifically, we theorized that ambiguity might elicit concerns about the risk of punishing unfairly, given that in one possible state of the world a norm violation has not actually occurred. Our research provides first evidence for these concerns in the 3PPG under ambiguity; yet, some inconsistencies in our findings question whether they affect 3PP.

On one hand, in Studies 1 to 3 and 4b, we observed that, under ambiguity, third parties with high Observer JS reduced their 3PP more pronouncedly than those with low Observer JS. This moderating effect did not replicate in Studies 4 and 5. Although we only observed this interaction effect in four out of six studies, it is important to reflect on why stronger justice concerns might lead third parties to refrain from punishing an ambiguous norm violation. A theoretically plausible explanation could relate to the aforementioned risk of punishing unfairly.

People may refrain from punishing an ambiguous norm violation given the possibility that the norm violation has not actually occurred and that punishment is consequently unjustified. This notion received tentative support from Studies 4 and 4b, where third parties had the opportunity to resolve the ambiguity of the norm violation (in Study 4, by incurring additional costs) and, hence, to exclude that punishment was unfair. Although Observer JS positively correlated with concerns about punishing unfairly (see Supplemental Tables S14–S16), we unexpectedly did not find it to predict the decision to resolve the ambiguity. However, what we did observe was that third parties who resolved the ambiguity reported being relatively more concerned about the possibility of punishing unfairly; therefore, resolving the ambiguity presumably offered them a means to alleviate these concerns. In fact, once they had resolved the ambiguity, their 3PP matched (Study 4b) or even exceeded (Study 4) the baseline 3PP observed under no ambiguity. While these findings speak in favor of people experiencing and addressing these concerns about punishing unfairly by resolving the ambiguity, we did not consistently observe them to mediate the effect of ambiguity on 3PP (only in Study 4b, but not in Studies 4 and 5).

As a second plausible mechanism, we considered that ambiguity might provide a situational justification or “moral wiggle room” for those who intend to remain passive, and thus, avoid incurring costs (Dana et al., 2007; Stüber, 2020). Our research does not offer clear evidence in this direction. When Observer JS moderated the effect of ambiguity (Studies 1–3 and 4b), we also observed that people with low Observer JS—thus, more prone to weight in the costs of 3PP (Lotz, Baumert, et al., 2011)—reduced their 3PP under ambiguity. However, this was to a lesser extent than those with high Observer JS. Furthermore, in Study 4, people who did not resolve the ambiguity reported a heightened concern about avoiding costs and showed low 3PP, but this could be because our experimental design incentivized to keep the ambiguity. Our mediational analyses did not show ambiguity to induce higher cost avoidance, although the latter negatively predicted 3PP.

Notwithstanding the mixed evidence regarding the theorized mechanisms, the present research undoubtedly identified a situational boundary of 3PP in the lab—namely, the ambiguity of the norm violation—that could clarify when 3PP occurs in real-life settings. At present, we cannot ascertain that our findings would generalize to the field; however, we suspect that the role of ambiguity might be crucial in accounting for the already discussed discrepancies between lab and field studies (Guala, 2012; Pedersen et al., 2018). Ambiguity should be a relevant explanatory factor of 3PP in contexts where situational and normative information is scarce and/or contradictory. For example, previous research in the field has showed that conflicting (hence, arguably “ambiguous”) injunctive and descriptive anti-littering norms discouraged third parties from punishing litterers (Berger & Hevenstone, 2016). Further research should offer systematic comparisons between lab and field experiments to corroborate that ambiguity certainly explains 3PP in more externally valid settings.

### Limitations and Future Research

The present findings and the discussed inconsistencies might serve as a starting point for future research.

First, the moderating role of Observer JS did not replicate consistently across studies. Our examination of methodological differences between the studies that showed the Ambiguity × Observer JS interaction and those that did not (e.g., use of the strategy method, sample demographics), did not clarify the inconsistent replicability of this interaction. Further research could help identify systematic differences that convincingly clarified this issue.

Second, the inconsistent observation of the indirect effect of ambiguity on 3PP through type I error concerns could be due to the post hoc assessment of behavioral motives. Although this type of measurement is common in psychological research (e.g., Eriksson et al., 2017), it has the important limitation of hinging on accurate introspection. This might make it difficult to capture nuanced motives and even offer confounded measurements, especially among participants less familiar with experimental settings (e.g., economic lab students in Study 4b vs. online panelists in Studies 4 and 5). For instance, by measuring type I error concerns after the punishment decision, some participants might have reported concerns about punishing unfairly that arose in reaction to their already exerted punishment—presumably correlating positively, instead of negatively, with 3PP and leading to an overall null effect. Yet, these limitations should not lead us to disregard the informative value of this type of measurement completely. Our post hoc questionnaire offered tentative evidence that, together with our findings on the resolution of ambiguity, may inform future research about the role of the theorized mechanisms.

Third, Study 5 suggested an alternative explanation to why ambiguity decreased 3PP, namely that, under ambiguity, people may simply not perceive the norm violation, especially when the latter is potentially less severe (in our case, in the *low expected value* conditions). Specifically, third parties could just be inattentive and miss the situation under ambiguity or, instead, struggle to disentangle whether a norm violation has occurred or not due to the ambiguous information. In the second case, one could still expect third parties to experience concerns about punishing unfairly, which might affect the decision to punish. Future research should address this nuanced distinction.

Finally, research on 3PP has been criticized for the presence of strong demand effects (Pedersen et al., 2018). For example, some have argued that the use of the strategy method enhances these demand effects (Pedersen et al., 2013); yet, we replicated the effect of ambiguity in setups other than the strategy method (Studies 4–5). Moreover, demand effects could result from the limited behavioral options of lab experiments (Pedersen et al., 2018). We provided participants with the opportunity to compensate the victim as an alternative behavioral reaction to counter this potential limitation. Still, we acknowledge that our design does not fully resemble the behavioral repertoire that third parties might have in real-life situations. Therefore, we reemphasize the need to replicate the present findings in the field to rule out the potential influence of demand effects inherent to settings with higher experimental control.

## Conclusion

The present research has taken an important step by establishing ambiguity of the norm violation as a critical situational boundary of 3PP. We demonstrated that, when a norm violation became ambiguous, third parties consistently punished less. When facing an ambiguous norm violation, an important consideration that could ultimately prevent some third parties from punishing is to avoid engaging in unfair punishment. When possible, third parties could overcome this concern of punishing unfairly by means of resolving the ambiguity. We showed that these concerns are indeed present under ambiguity, but it is unclear whether they prevent 3PP. Moreover, our findings emphasize that the estimation of the prevalence of 3PP and other types of behavior can improve by considering factors like situational ambiguity, which likely characterize many everyday situations outside the lab.

## Supplemental Material

sj-docx-1-psp-10.1177_01461672211067675 – Supplemental material for “Proof *Under* Reasonable Doubt”: Ambiguity of the Norm Violation as Boundary Condition of Third-Party Punishment Click here for additional data file. Supplemental material, sj-docx-1-psp-10.1177_01461672211067675 for “Proof *Under* Reasonable Doubt”: Ambiguity of the Norm Violation as Boundary Condition of Third-Party Punishment by Daniel Toribio-Flórez, Julia Saße and Anna Baumert in Personality and Social Psychology Bulletin

## Notes

1. In Studies 1 to 3, 1 ECU = €1.00.

2. In Studies 4 to 5, 1 ECU = €0.50.

3. In the postexperimental questionnaire of Studies 4 and 4b, we asked participants in the ambiguity conditions to report their assumptions about Person A’s endowments probability distribution (see Supplemental Figure S11). Roughly, 38% to 54% reported to assume a uniform distribution (i.e., “any amount from 2 to 10 was equally likely”), whereas 28% to 31% reported not to have made any assumption.
