# Supplementary material for: “Proof Under Reasonable Doubt”: Ambiguity of the Norm Violation as Boundary Condition of Third-Party Punishment
Source: Pers Soc Psychol Bull. 2022 Feb 1;49(3):429–46. doi: 10.1177/01461672211067675 (PMC9900685; doi:10.1177/01461672211067675)
Supplement: sj-docx-1-psp-10.1177_01461672211067675 – Supplemental material for “Proof Under Reasonable Doubt”: Ambiguity of the Norm Violation as Boundary Condition of Third-Party Punishment [file sj-docx-1-psp-10.1177_01461672211067675.docx]

**Supplement**

Table of Contents

[1. Results on Third-Party Compensation 2](#_Toc89095268)

[Studies 1-3 2](#_Toc89095269)

[Study 4 4](#_Toc89095270)

[Study 5 5](#_Toc89095271)

[2. Deviations from Preregistrations 6](#_Toc89095272)

[Uncertainty of Intervention Costs 6](#_Toc89095273)

[Dichotomous vs. Continuous Measures of Punishment and Compensation 13](#_Toc89095274)

[3. Levels of Punishment and Compensation in Single Decisions of Strategy Method – Studies 1-3 16](#_Toc89095275)

[4. Study 4b – Method, Results and Discussion 17](#_Toc89095276)

[5. Power Analyses 24](#_Toc89095277)

[Study 3 24](#_Toc89095278)

[Studies 4 and 4b 24](#_Toc89095279)

[6. Elicitation of Dictator Decisions in Studies 4, 4b and 5 26](#_Toc89095280)

[7. Post-Experimental Questionnaire – Studies 4, 4b and 5 27](#_Toc89095281)

[Complete List of Items Presented in each Study 27](#_Toc89095282)

[Bivariate Correlations between Post-Experimental Questionnaire, JS, and SVO 29](#_Toc89095283)

[Participants’ Assumptions about Person A’s Endowment under Ambiguity 30](#_Toc89095284)

[8. Study 5 – Secondary Analyses 31](#_Toc89095285)

[Analyses with Preregistered Items of Type I Error 31](#_Toc89095286)

[SVO and Inequality Aversion 32](#_Toc89095287)

[OJS and SVO interactions with Ambiguity and Expected Value. 34](#_Toc89095288)

[9. References 35](#_Toc89095289)

# Results on Third-Party Compensation

## Studies 1-3

| **Table S1**  *Descriptive Statistics of Compensation per Condition - Studies 1-3.* | | | | | | |
| --- | --- | --- | --- | --- | --- | --- |
|  | **Punishment** | | | | | |
|  | *M* | *SD* | | | *Perc.* (%) | |
| **Study 1** |  |  | | |  |  |
| No Ambiguity | 13.11 | 7.56 | | | 92.90 | |
| Ambiguity | 9.42 | 6.83 | | | 89.82 | |
| **Study 2** |  |  |  |  |  |  |
| No Ambiguity | 12.26 | 7.94 | | | 88.57 | |
| Ambiguity | 8.53 | 7.63 | | | 82.51 | |
| **Study 3** |  |  |  |  |  |  |
| No Ambiguity | 8.29 | 7.96 | | | 69.40 | |
| Ambiguity | 6.10 | 7.16 | | | 66.25 | |
| *Note.* Compensation = Amount of ECUs (1 ECU = 1 Euro) added to Person B. *M* and *SD* = mean and standard deviation of the sum of Euros compensated across decisions to unequal splits from Person A (i.e., €[0 to 4] coins) to Person B. *Perc*. (%) = percentage of participants who compensated at least 1 ECU. | | | | | | |

| **Table S2**  *Tested Multilevel Model on Compensation in Studies 1-3.* | | | | | | | | | | | | |
| --- | --- | --- | --- | --- | --- | --- | --- | --- | --- | --- | --- | --- |
|  |  | **Study 1** | | |  | **Study 2** | | |  | **Study 3** | | |
| **Parameters** |  | **β** [95% CI] | ***t*** | ***p*** |  | **β** [95% CI] | ***t*** | ***p*** |  | **β** [95% CI] | ***t*** | ***p*** |
| Ambiguity of Norm Violation |  | -.50 [-.60, -.40] | -10.10 | **<.001***** |  | -.46 [-.53, -.39] | -12.51 | **<.001***** |  | -.29 [-.34, -.24] | -10.72 | **<.001***** |
| Perpetrator JS |  | .10 [-.04, .24] | 1.44 | .152 |  | .15 [.02, .28] | 2.34 | **.020**** |  | .24 [.11, .37] | 3.72 | **<.001***** |
| Observer JS |  | .14 [.00, .28] | 1.99 | **.048*** |  | .13 [.00, .25] | 1.95 | .052 |  | .09 [-.03, .22] | 1.46 | .146 |
| Ambiguity x Perpetrator JS |  | .04 [-.06, .14] | 0.80 | .426 |  | -.01 [-.08, .07] | -0.13 | .898 |  | -.03 [-.09, .03] | -0.89 | .375 |
| Ambiguity x Observer JS |  | -.07 [-.17, -.03] | -1.30 | .193 |  | -.04 [-.12, .04] | -0.95 | .343 |  | -.09 [-.15, -.02] | -2.63 | **.009**** |
| **Random Effects** |  |  |  |  |  |  |  |  |  |  |  |  |
| σ^2^ |  | 21.88 | | |  | 19.29 | | |  | 12.07 | | |
| τ_00_ _ID_ |  | 28.16 | | |  | 39.00 | | |  | 41.51 | | |
| ICC _ID_ |  | 0.56 | | |  | 0.67 | | |  | 0.77 | | |
| N _ID_ |  | 162 | | |  | 221 | | |  | 283 | | |
| Observations |  | 648 | | |  | 884 | | |  | 1125 | | |
| Marginal / Conditional R^2^ |  | 0.097 / 0.605 | | |  | 0.100 / 0.702 | | |  | 0.090 / 0.795 | | |
| *Note*. JS = Justice Sensitivity, σ^2^ = Residual variance; τ_00_ _ID_ = Variance of the intercept; ICC _ID_ = Intraclass correlation coefficient; N _ID_ = Total number of individuals. *** *p* < .001, ** *p* < .01, * *p* < .05. | | | | | | | | | | | | |

| **Table S3**  *ANOVA Table of Multilevel Model on Compensation Accounting for Order Effects in Study 3.* | | | | |
| --- | --- | --- | --- | --- |
|  | **Compensation** | | | |
| **Parameters** | ***df*** | ***F*** | ***p*** | **η_p_^2^** |
| Ambiguity of Norm Violation | 1, 824 | 111.57 | < .001 | .117 |
| Perpetrator JS | 1, 265 | 16.02 | < .001 | .019 |
| Observer JS | 1, 265 | 0.03 | .870 | .000 |
| Position | 1, 824 | 0.79 | .376 | .001 |
| Ambiguity Order | 5, 265 | 0.83 | .531 | .005 |
| Ambiguity x Perpetrator JS | 1, 824 | 0.20 | .657 | .000 |
| Ambiguity x Observer JS | 1, 824 | 7.43 | .007 | .009 |
| Ambiguity x Ambiguity Order | 5, 824 | 1.27 | .275 | .007 |
| Perpetrator JS x Ambiguity Order | 5, 265 | 1.48 | .197 | .009 |
| Observer JS x Ambiguity Order | 5, 265 | 1.39 | .227 | .008 |
| Ambiguity x Perpetrator JS x Ambiguity Order | 5, 824 | 1.75 | .120 | .010 |
| Ambiguity x Observer JS x Ambiguity Order | 5, 824 | 1.33 | .249 | .008 |
| *Note*. JS = Justice Sensitivity, *df* = Numerator and denominator degrees of freedom calculated with Satterthwaite’s method. | | | | |

## Study 4

| **Table S4**  *Tested Multiple Regression Model on Compensation in Study 4.* | | | | |
| --- | --- | --- | --- | --- |
| **Parameters** |  | **β** [95% CI] | ***t*** | ***p*** |
| Ambiguity of Norm Violation |  | -.20 [-.41, .01] | -1.87 | .062 |
| Perpetrator JS |  | .08 [-.10, .25] | 0.88 | .382 |
| Observer JS |  | -.04 [-.22, .14] | -0.46 | .646 |
| Ambiguity x Perpetrator JS |  | .08 [-.16, .33] | 0.67 | .501 |
| Ambiguity x Observer JS |  | -.08 [-.32, .17] | -0.61 | .539 |
| Observations |  | 345 | | |
| R^2^ / Adj. R^2^ |  | .023 / .009 | | |
| *Note*. *** *p* < .001, ** *p* < .01, * *p* < .05. | | | | |

**Figure S1**

*
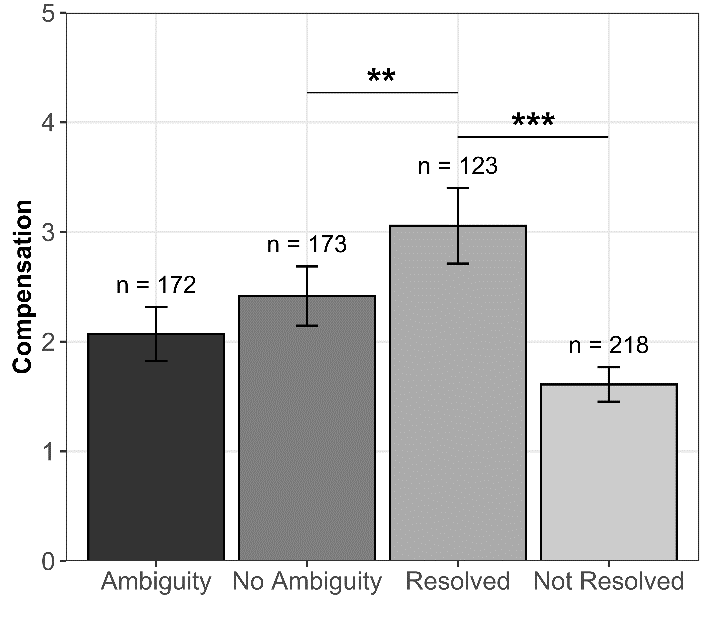
Mean Levels of Compensation across experimental conditions in Study 4. 95% CIs error bars.*

*Note.* Compensation captured the total amount of ECUs that participants wished to add to Person B (from 0 to 10 ECUs).

*** *p* < .001, ** *p* < .01. 95% CIs error bars.

## Study 5

| **Table S5**  *Tested Multiple Regression Model on Compensation in Study 5.* | | | | |
| --- | --- | --- | --- | --- |
| **Parameters** |  | **β** [95% CI] | ***t*** | ***p*** |
| Ambiguity of Norm Violation |  | -.07 [-.27, .12] | -0.73 | .465 |
| Expected Value |  | .04 [-.16, .24] | 0.37 | .711 |
| Ambiguity x Expected Value |  | -.14 [-.42, .14] | -0.99 | .322 |
| Observations |  | 778 | | |
| R^2^ / Adj. R^2^ |  | .007 / .003 | | |
| *Note*. *** *p* < .001, ** *p* < .01, * *p* < .05. | | | | |

**Figure S2**


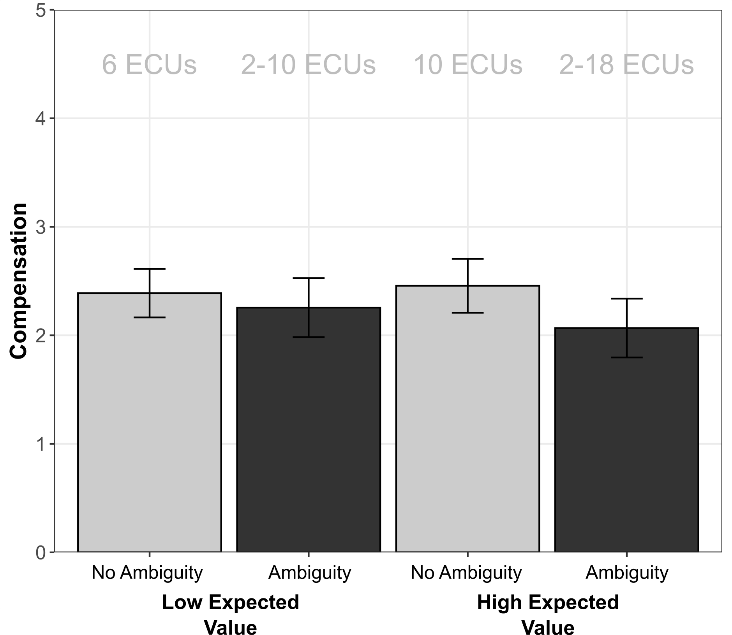
*Mean Levels of Compensation across experimental conditions in Study 5.*

*Note.* Compensation captured the total amount of ECUs that participants wished to add to Person B (from 0 to 10 ECUs).

95% CIs error bars.

# Deviations from Preregistrations

## Uncertainty of Intervention Costs

As mentioned in the main manuscript and the preregistrations of Studies 1 and 2, the design of these studies included a second experimental manipulation intended to address whether uncertain intervention costs hindered 3PP. In what follows, we provide a brief introduction of the theoretical background behind this manipulation and justify why we decided it not to be part of the main manuscript.

**Theoretical Background.** In everyday life, under situational ambiguity, third parties might not know a priori the exact costs of engaging into 3PP. To our knowledge, this situational aspect has been neglected in research on 3PP, where experimenters generally establish common knowledge about some fixed cost associated with any third-party intervention. We therefore aimed to investigate whether an uncertain level of intervention costs influenced the decision of exerting 3PP.

Similarly to how people overestimate the probability of extreme life events (Lichtenstein et al., 1978), we believed that a situation where costs are uncertain could lead third parties to overestimate, and therefore attribute more subjective weight to, potential high costs, even when these are unlikely (Barberis, 2013; Rozin & Royzman, 2001; Tversky & Kahneman, 1992). This overestimation of uncertain intervention costs would imply an increase of the overall cost-benefit ratio of the behavior, which, in turn, could plausibly hinder any intention of engaging in 3PP (Egas & Riedl, 2008). Thus, we predicted that introducing uncertain (vs. fixed) levels of intervention costs would decrease 3PP.

In addition, we expected that the impact of uncertain costs would differ depending on a third facet of Justice Sensitivity (JS). More specifically, Beneficiary JS captures dispositional concerns to perceive and react against injustice from the perspective of a passive *beneficiary* of the unjust situation (Baumert & Schmitt, 2016). We thought that Beneficiary JS would be relevant to predict interindividual differences in the reaction to uncertain costs. Avoiding own costs in the light of another person being unfairly disadvantaged could be construed as a kind of indirect benefit from the situation. People high in Beneficiary JS should be motivated to avoid this kind of benefit, and therefore accept uncertain costs to punish unfairness, whereas people low in Beneficiary JS should lack this motivation and be reluctant to incur uncertain and thus potentially high costs. Thus, we expected (and preregistered) that those high in Beneficiary JS should show similar levels of costly third-party punishment regardless of the (un-)certainty of intervention costs, whereas those low in Beneficiary JS should punish less when intervention costs are uncertain.

**Method and Results.** As described in the main manuscript, two of the rounds of the 3PPG introduced uncertainty of intervention costs for Person C. In the *low uncertainty* conditions, modifying the outcome of Person A or Person B by €1 entailed a fixed associated cost of ½ ECUs for Person C. In the *high uncertainty* condition, the cost for Person C for modifying Person A’s or B’s outcome by 1 ECU was determined at random in a range from 0.01 to 1 ECUs, assuming a uniform probability distribution. Participants did not receive information about the underlying distribution. Thus, under high uncertainty, participants in the role of Person C did not know the amount of costs that they would incur.

Table S6 presents levels of costly third-party punishment and compensation in Studies 1 to 3 across the different rounds of the 3PPG (in this case, splitting also between rounds including certain vs. uncertain intervention costs). Table S7 to S9 summarize the different preregistered multilevel models on 3PP from Studies 1 to 3, including Model 1 and 3 where uncertainty of intervention costs was entered as a Level-1 fixed factor. In none of the studies, we observed a significant effect of uncertainty of intervention costs, nor a significant interaction with Beneficiary JS. Important for the main manuscript, uncertainty of intervention costs did not affect the main effect of ambiguity of the norm violation, as Model 1 indicated when including the interaction term between Ambiguity and Uncertainty – in Study 1, β = .00, *t*(482) = -0.02, *p* = .982, 95%CI [-.20, .19], in Study 2, β = .02, *t*(666) = 0.21, *p* = .837, 95%CI [-.13, .16], and in Study 3, β = .04, *t*(826) = 0.54, *p* = .586, 95%CI [-.10, .17].

| **Table S6**  *Descriptive Statistics for Punishment and Compensation in the 3PPG for Each Condition across the Different Studies.* | | | | | | | |
| --- | --- | --- | --- | --- | --- | --- | --- |
|  |  | **Punishment** |  |  |  | **Compensation** |  |
|  | *M*_Sum_ | *SD*_Sum_ | *Perc.* (%) |  | *M*_Sum_ | *SD*_Sum_ | *Perc.* (%) |
| **Study 1** |  |  |  |  |  |  |  |
| Low Ambiguity + Low Uncertainty | 10.72 | 7.50 | 85.28 |  | 13.42 | 7.59 | 93.25 |
| Low Ambiguity + High Uncertainty | 11.00 | 7.94 | 83.95 |  | 12.82 | 7.49 | 92.64 |
| High Ambiguity + Low Uncertainty | 5.99 | 6.14 | 73.01 |  | 9.47 | 7.01 | 88.34 |
| High Ambiguity + High Uncertainty | 6.25 | 5.95 | 77.91 |  | 9.36 | 6.62 | 91.41 |
| **Study 2** |  |  |  |  |  |  |  |
| Low Ambiguity + Low Uncertainty | 9.89 | 7.51 | 79.82 |  | 12.69 | 7.98 | 89.69 |
| Low Ambiguity + High Uncertainty | 9.48 | 8.06 | 74.44 |  | 11.82 | 7.89 | 87.44 |
| High Ambiguity + Low Uncertainty | 5.10 | 6.35 | 65.92 |  | 8.83 | 7.88 | 82.06 |
| High Ambiguity + High Uncertainty | 4.81 | 6.05 | 65.02 |  | 8.23 | 7.38 | 82.95 |
| **Study 3** |  |  |  |  |  |  |  |
| Low Ambiguity + Low Uncertainty | 6.58 | 7.16 | 61.15 |  | 8.62 | 8.14 | 71.17 |
| Low Ambiguity + High Uncertainty | 6.27 | 7.48 | 57.76 |  | 7.95 | 7.78 | 67.62 |
| High Ambiguity + Low Uncertainty | 3.40 | 4.95 | 53.62 |  | 6.22 | 7.36 | 65.25 |
| High Ambiguity + High Uncertainty | 3.31 | 5.09 | 51.62 |  | 5.98 | 6.96 | 67.26 |
| *Note.* Punishment = Amount of Euro subtracted from Person A. Compensation = Amount of Euro added to Person B. *M*_Sum_ and *SD*_Sum_ = mean and standard deviation of the sum of Euros punished or compensated across decisions to unequal splits from Person A (i.e., €[0 to 4] coins to Person B). *Perc*. = percentage of participants who punished or compensated at least €1. | | | | | | | |

| **Table S7**  *Tested Multilevel Models on Punishment in Study 1.* | | | | | | | | | |
| --- | --- | --- | --- | --- | --- | --- | --- | --- | --- |
|  | **Model 1** | | | **Model 2** | | | **Model 3** | | |
| **Parameter** | **β** [95% CI] | ***t*** | ***p*** | **β** [95% CI] | ***t*** | ***p*** | **β** [95% CI] | ***t*** | ***p*** |
| **Fixed Effects** | | | | | | | | | |
| Ambiguity of norm violation | -.65  [-.74 – -.55] | -13.07 | <.001 | -.65  [-.74 – -.55] | -13.12 | <.001 | - | - | - |
| Uncertainty of intervention costs | .04  [-.06 – .13] | 0.71 | .478 | - | - | - | .03 [-.08 – .14] | 0.51 | .607 |
| Perpetrator JS | - | - | - | .06  [-.07 – .20] | 0.91 | .364 | - | - | - |
| Observer JS | - | - | - | .13  [.00 – .27] | 1.90 | .058 | - | - | - |
| Beneficiary JS | - | - | - | - | - | - | .10  [-.04 – .23] | 1.44 | .150 |
| Ambiguity X Perpetrator JS | - | - | - | .02  [-.09 – .12] | 0.31 | .755 | - | - | - |
| Ambiguity X Observer JS | - | - | - | -.14  [-.24 – -.04] | -2.66 | .008 | - | - | - |
| Uncertainty X Beneficiary JS | - | - | - | - | - | - | -.04 [-.16 – .07] | -0.74 | .459 |
| **Random Effects** | | | | | | | | | |
| σ^2^ | 21.18 | | | 20.99 | | | 28.68 | | |
| τ_00_ _ID_ | 27.06 | | | 27.04 | | | 25.88 | | |
| ICC _ID_ | 0.56 | | | 0.56 | | | 0.47 | | |
| N _ID_ | 163 | | | 162 | | | 159 | | |
| Observations | 648 | | | 644 | | | 632 | | |
| Marginal R^2^ / Conditional R^2^ | 0.104 / 0.607 | | | 0.119 / 0.615 | | | 0.007 / 0.478 | | |
| *Note*. JS = Justice Sensitivity, σ^2^ = Residual variance; τ_00_ _ID_ = Variance of the intercept; ICC _ID_ = Intraclass correlation coefficient; N _ID_ = Total number of individuals. | | | | | | | | | |

| **Table S8**  *Tested Multilevel Models on Punishment in Study 2.* | | | | | | | | | |
| --- | --- | --- | --- | --- | --- | --- | --- | --- | --- |
|  | **Model 1** | | | **Model 2** | | | **Model 3** | | |
| **Parameter** | **β** [95% CI] | ***t*** | ***p*** | **β** [95% CI] | ***t*** | ***p*** | **β** [95% CI] | ***t*** | ***p*** |
| **Fixed Effects** | | | | | | | | | |
| Ambiguity of norm violation | -0.64 [-0.71 – -0.57] | -17.39 | <0.001 | -0.63  [-0.70 – -0.56] | -17.30 | < .001 | - | - | - |
| Uncertainty of intervention costs | -0.05 [-0.12 – 0.02] | -1.29 | 0.196 | - | - | - | -0.04  [-0.13 – 0.05] | -0.86 | 0.389 |
| Perpetrator JS | - | - | - | -0.03  [-0.16 – 0.09] | -0.53 | 0.594 | - | - | - |
| Observer JS | - | - | - | 0.15 [0.02 – 0.27] | 2.32 | 0.021 | - | - | - |
| Beneficiary JS | - | - | - | - | - | - | 0.05  [-0.06 – 0.17] | 0.89 | 0.374 |
| Ambiguity X Perpetrator JS | - | - | - | 0.05  [-0.03 – 0.13] | 1.15 | 0.250 | - | - | - |
| Ambiguity X Observer JS | - | - | - | -0.12  [-0.20 – -0.04] | -2.95 | 0.003 | - | - | - |
| Uncertainty X Beneficiary JS | - | - | - | - | - | - | 0.01  [-0.08 – 0.11] | 0.20 | . 0.842 |
| **Random Effects** | | | | | | | | | |
| σ^2^ | 16.48 | | | 16.33 | | | 23.94 | | |
| τ_00_ _ID_ | 33.04 | | | 33.23 | | | 31.69 | | |
| ICC _ID_ | 0.67 | | | 0.67 | | | 0.57 | | |
| N _ID_ | 223 | | | 221 | | | 216 | | |
| Observations | 892 | | | 884 | | | 864 | | |
| Marginal / Conditional R^2^ | 0.102 / 0.701 | | | 0.110 / 0.707 | | | 0.004 / 0.571 | | |
| *Note*. JS = Justice Sensitivity, σ^2^ = Residual variance; τ_00_ _ID_ = Variance of the intercept; ICC _ID_ = Intraclass correlation coefficient; AIC = Akaike Information Criterion. | | | | | | | | | |

| **Table S9**  *Tested Multilevel Models on Punishment in Study 3.* | | | | | | | | | |
| --- | --- | --- | --- | --- | --- | --- | --- | --- | --- |
|  | **Model 1** | | | **Model 2** | | | **Model 3** | | |
| **Parameter** | **β** [95% CI] | ***t*** | ***p*** | **β** [95% CI] | ***t*** | ***p*** | **β** [95% CI] | ***t*** | ***p*** |
| **Fixed Effects** | | | | | | | | | |
| Ambiguity of norm violation | -.48  [-.54, -.41] | -14.208 | <0.001 | -.48  [-.54, -.41] | -14.553 | <0.001 | - | - | - |
| Uncertainty of intervention costs | -.03  [-.10, .04] | -0.886 | .376 | - | - | - | -.03  [-.10, .04] | -0.849 | 0.396 |
| Perpetrator JS | - | - | - | .19  [.06, .31] | 2.965 | 0.003 | - | - | - |
| Observer JS | - | - | - | .11  [-.02, .23] | 1.704 | 0.089 | - | - | - |
| Beneficiary JS | - | - | - | - | - | - | .20  [.10, .31] | 3.887 | <0.001 |
| Ambiguity X Perpetrator JS | - | - | - | -.11  [-.18, -.03] | -2.669 | 0.008 | - | - | - |
| Ambiguity X Observer JS | - | - | - | -.11  [-.19, -.03] | -2.733 | 0.006 | - | - | - |
| Uncertainty X Beneficiary JS | - | - | - | - | - | - | -.03  [-.11, .04] | -0.915 | 0.361 |
| **Random Effects** | | | | | | | | | |
| σ^2^ | 13.01 | | | 12.55 | | | 16.17 | | |
| τ_00_ _ID_ | 26.25 | | | 25.39 | | | 24.12 | | |
| ICC _ID_ | 0.67 | | | 0.67 | | | 0.60 | | |
| N _ID_ | 281 | | | 281 | | | 281 | | |
| Observations | 1108 | | | 1108 | | | 1108 | | |
| Marginal / Conditional R^2^ | 0.057 / 0.688 | | | 0.094 / 0.700 | | | 0.036 / 0.613 | | |
| *Note*. JS = Justice Sensitivity, σ^2^ = Residual variance; τ_00_ _ID_ = Variance of the intercept; ICC _ID_ = Intraclass correlation coefficient; AIC = Akaike Information Criterion. | | | | | | | | | |

**Discussion.** Despite the relevant theoretical role that uncertainty of intervention costs could have for 3PP, our manipulation did not seem to exert any effect on punishment. There are different potential explanations for these null findings. For example, if one assumed that individuals were risk-neutral, standard economic theory would predict the observed null effect given that the expected value is equal across uncertainty conditions (i.e., 0.5 ECUs). A second alternative refers to large interindividual differences in risk or uncertainty tolerance, which could have triggered opposing reactions to our manipulation (i.e., with some participants being very risk-seeking and other participants being very risk-averse) and have resulted in an overall null effect. Lacking of any measure of dispositional risk tolerance, we cannot test if this was the case, but a visual inspection of the distribution of 3PP across experimental conditions did not seem to support such a bimodal trend. Our main suspicion, however, is that these null findings are due to the manipulation of cost uncertainty itself, which was perhaps insufficiently strong. In particular, we believe that the range in which costs could vary (0.01 to 1 ECUs) did not resemble the potential, yet unlikely, extreme costs that we argued could discourage 3PP in contexts of high situational ambiguity. In other words, the highest possible cost (i.e., a maximum of 1 ECU per 1 ECU punished or compensated) might not have had enough subjective value for people to discourage intervention. However, this would be in contrast to previous work showing that a 1:1 cost-benefit ratio already has detrimental effects, at least on second-party punishment (Egas & Riedl, 2008).

Whatever the case may be, we suggest that future attempts for examining the impact of the costs uncertainty, and the impact of intervention costs more generally, could be interesting for a deeper comprehension of 3PP and its boundaries. With regard to our manuscript, the consistent null results and null influence on our main findings regarding ambiguity of the norm violation, made us decide not to include these results as part of the manuscript for a matter of conciseness, clarity and focus on the more informative findings.

## Dichotomous vs. Continuous Measures of Punishment and Compensation

In the preregistrations of Studies 1 and 2, we had included a third research question referring to the association between 3PP in the context of the 3PPG and bystander intervention (in Study 1) and intervention intention (in Study 2) against an embezzlement. In the case of Study 1, this embezzlement was staged in the lab during a second lab session, and reactions to this norm violation were video-coded. In Study 2, we measured intervention intention after describing the embezzlement situation in a vignette and video-vignette format.

In order to compare participants’ decisions in the 3PPG with the behavioral coding of their reaction to the embezzlement, we originally preregistered to use a dichotomous measure of costly punishment (0 – no punishment, 1 – punishment of at least 1 ECU) and analyze the effects of interest through logistic multilevel models.

However, this third research question exceeded the scope of the current manuscript. We therefore deviated from the preregistered analyses by using a continuous measure of 3PP, which certainly provided more statistical power and sensitivity to our analyses than the dichotomous measure would have done. In any case, the results from our preregistered logistic multilevel models for Studies 1 and 2 are presented in Tables S10 and S11, respectively, and closely resembled the results obtained with our continuous dependent measure.

| **Table S10**  *Multilevel Logistic Regression Models with Dichotomous Dependent Measure of Punishment in Study 1.* | | | | | | | | | |
| --- | --- | --- | --- | --- | --- | --- | --- | --- | --- |
|  | **Model 1** | | | **Model 2** | | | **Model 3** | | |
| **Parameter** | **Odds ratio** 95% CI | ***z*** | ***p*** | **Odds ratio** 95% CI | ***z*** | ***p*** | **Odds ratio** 95% CI | ***z*** | ***p*** |
| **Fixed Effects** | | | | | | | | | |
| Ambiguity of norm violation | 0.09  [0.03 – 0.23] | -4.94 | <0.001 | 0.06  [0.02 – 0.18] | -4.93 | <0.001 | - | - | - |
| Uncertainty of intervention costs | 1.52  [0.68 – 3.41] | 1.04 | 0.300 | - | - | - | 1.36  [0.65 – 2.83] | 0.81 | 0.418 |
| Perpetrator JS | - | - | - | 0.88  [0.27 – 2.84] | -0.21 | 0.833 | - | - | - |
| Observer JS | - | - | - | 2.38  [0.76 – 7.49] | 1.48 | 0.138 | - | - | - |
| Beneficiary JS | - | - | - | - | - | - | 1.34  [0.61 – 2.93] | 0.74 | 0.460 |
| AmbiguityXPerpetrator JS | - | - | - | 1.38  [0.62 – 3.05] | 0.79 | 0.432 | - | - | - |
| AmbiguityXObserver JS | - | - | - | 0.40  [0.18 – 0.90] | -2.22 | 0.026 | - | - | - |
| UncertaintyXBeneficiary JS | - | - | - | - | - | - | 0.73  [0.41 – 1.30] | -1.06 | 0.288 |
| Constant | 22242  [2965 – 166831] | 9.74 | <0.001 | 39168  [4466 – 343542] | 9.62 | <0.001 | 1895.34  [344 – 10449] | 8.67 | \| <0.001 \| \| --- \| \|  \| |
| **Random Effects** | | | | | | | | | |
| σ^2^ | 3.29 | | | 3.29 | | | 3.29 | | |
| τ_00_ _ID_ | 126.28 | | | 125.09 | | | 84.92 | | |
| ICC _ID_ | 0.97 | | | 0.97 | | | 0.96 | | |
| N _ID_ | 163 | | | 162 | | | 159 | | |
| Observations | 648 | | | 644 | | | 635 | | |
| Marginal R^2^ / Conditional R^2^ | 0.012 / 0.975 | | | 0.019 / 0.975 | | | 0.001 / 0.963 | | |
| *Note*. JS = Justice Sensitivity, σ^2^ = Residual variance; τ_00_ _ID_ = Variance of the intercept; ICC _ID_ = Intraclass correlation coefficient; N _ID_ = Total number of individuals; AIC = Akaike Information Criterion. | | | | | | | | | |

| **Table S11**  *Multilevel Logistic Regression Models with Dichotomous Dependent Measure of Punishment in Study 2.* | | | | | | | | | |
| --- | --- | --- | --- | --- | --- | --- | --- | --- | --- |
|  | **Model 1** | | | **Model 2** | | | **Model 3** | | |
| **Parameter** | **Odds ratio** 95% CI | ***z*** | ***p*** | **Odds ratio** 95% CI | ***z*** | ***p*** | **Odds ratio** 95% CI | ***z*** | ***p*** |
| **Fixed Effects** | | | | | | | | | |
| Ambiguity of norm violation | 0.06  [0.03 – 0.14] | -6.67 | <0.001 | 0.05  [0.02 – 0.13] | -6.43 | <0.001 | - | - | - |
| Uncertainty of intervention costs | 0.47  [0.24 – 0.91] | -2.24 | 0.025 | - | - | - | 0.65  [0.36 – 1.18] | -1.43 | 0.154 |
| Perpetrator JS | - | - | - | 0.70  [0.23 – 2.16] | -0.61 | 0.541 | - | - | - |
| Observer JS | - | - | - | 2.54  [0.88 – 7.35] | 1.72 | 0.085 | - | - | - |
| Beneficiary JS | - | - | - | - | - | - | 1.17  [0.59 – 2.30] | 0.44 | 0.658 |
| AmbiguityXPerpetrator JS | - | - | - | 1.64  [0.75 – 3.59] | 1.24 | 0.216 | - | - | - |
| AmbiguityXObserver JS | - | - | - | 0.50  [0.28 – 0.92] | -2.24 | 0.025 | - | - | - |
| UncertaintyXBeneficiary JS | - | - | - | - | - | - | 1.14  [0.75 – 1.73] | 0.60 | 0.550 |
| Constant | 55760  [8645 – 359658] | 11.49 | <0.001 | 40477  [6381 – 256740] | 11.26 | <0.001 | 2422.51  [508.77 – 11534.71] | 9.79 | <0.001 |
| **Random Effects** | | | | | | | | | |
| σ^2^ | 3.29 | | | 3.29 | | | 3.29 | | |
| τ_00_ _ID_ | 164.63 | | | 163.64 | | | 105.11 | | |
| ICC _ID_ | 0.98 | | | 0.98 | | | 0.97 | | |
| N _ID_ | 223 | | | 221 | | | 216 | | |
| Observations | 892 | | | 884 | | | 864 | | |
| Marginal R^2^ / Conditional R^2^ | 0.013 / 0.981 | | | 0.016 / 0.981 | | | 0.001 / 0.970 | | |
| *Note*. JS = Justice Sensitivity, σ^2^ = Residual variance; τ_00_ _ID_ = Variance of the intercept; ICC _ID_ = Intraclass correlation coefficient; N _ID_ = Total number of individuals; AIC = Akaike Information Criterion. | | | | | | | | | |

# Levels of Punishment and Compensation in Single Decisions of Strategy Method – Studies 1-3

**Figure S3**

*
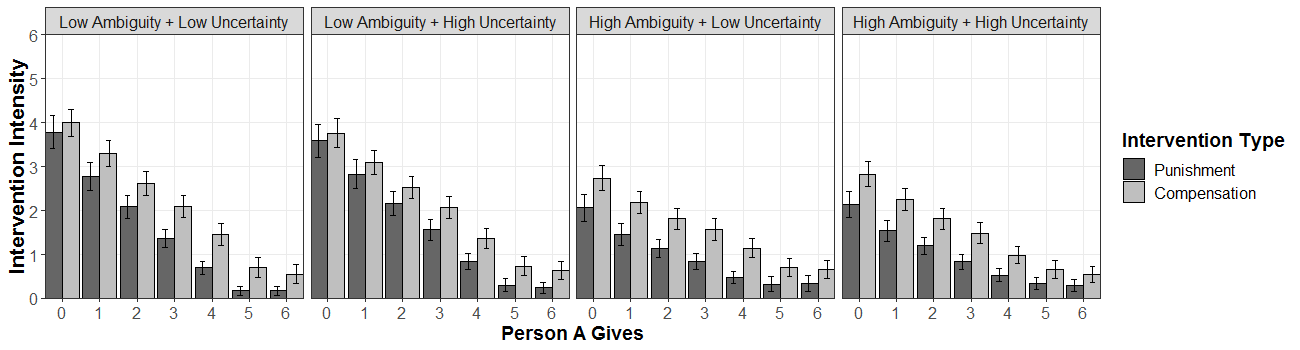
Mean Levels of Punishment and Compensation in Single Decisions of Strategy Method, Separate for Rounds. Error bars 95% CIs – Study 1.*

**Figure S4**

*Mean Levels of Punishment and Compensation in Single Decisions of Strategy Method, Separate for Rounds. Error bars 95% CIs – Study 2.*


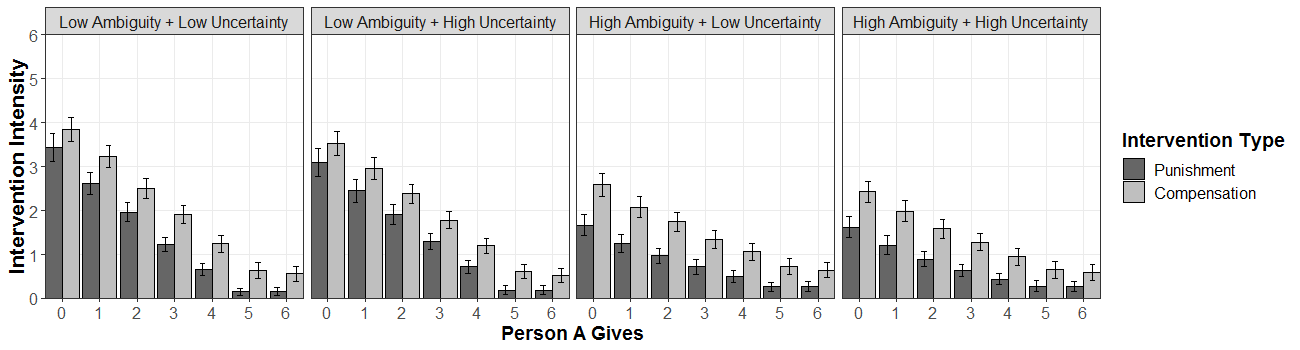


**Figure S5**

***
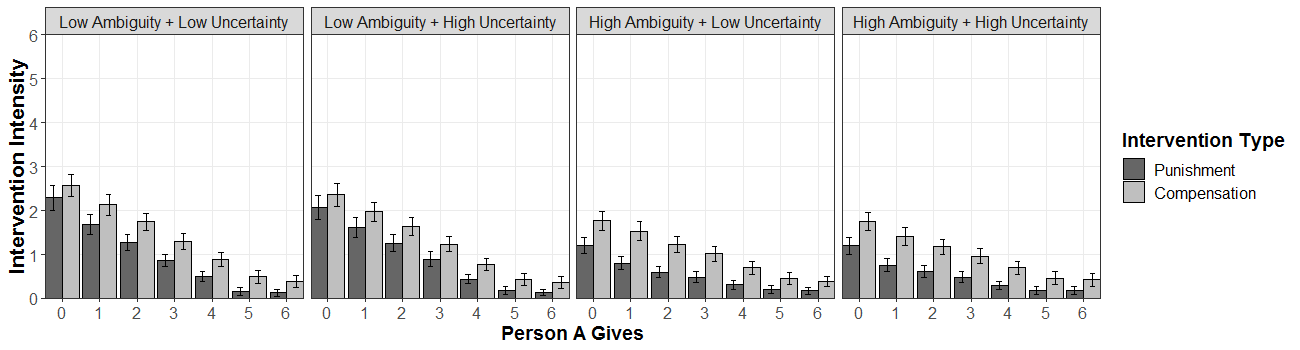
****Mean* *Levels of Punishment and Compensation in Single Decisions of Strategy Method, Separate for Rounds. Error bars 95% CIs – Study 3.*

# Study 4b – Method, Results and Discussion

Study 4b was similar to Study 4, with the only difference that in the *resolve/not resolve* condition, the decision to resolve the ambiguity by revealing Person A’s endowment was cost-free.

We tested the same preregistered hypotheses (H3-H6) and administered a similar post-experimental questionnaire to gauge different considerations that third parties might have had when deciding a) whether to resolve the ambiguity and b) whether to punish.

**Method**

***Participants***

Based on the same power analysis used in Study 4 (for details, see Section 5), we recruited 711 participants. We excluded data of 8 who did not finish the study and 70 who failed preregistered comprehension checks about the 3PPG. Therefore, the resulting sample was 633 participants, mainly undergraduate students from diverse disciplines (59% women; age range from 18 to 66, *M* = 25.47, *SD* = 5.51). They received a fixed monetary reward of €2.00 and they could additionally earn up to €5.00 in the 3PPG.

***Procedure***

In Study 4b, there was no lottery-voucher that participants could earn at the end of the study. Therefore, in the *resolve/not resolve* condition, participants could decide to resolve the ambiguity or not without incurring an additional cost.

Any other detail about the procedure was identical to Study 4.

***Design, Measures, and Statistical Analyses***

The design, measures, and performed statistical analyses were identical to Study 4.

***Post-experimental Questionnaire***

The items in the post-experimental questionnaire intended to capture type I error concerns and cost avoidance were the same as in Study 4 (see Table S13, in pp. 27-28).

**Main Results**

Comparing the *no* *ambiguity* and the *ambiguity* conditions, we observed that 3PP was significantly lower in the latter than in the former – supporting H1 (see Table S12, Model 1a). Furthermore, we observed a significant positive effect of Observer JS, but not Perpetrator JS. The two-way interactions were not significant. However, in a model without Perpetrator JS, the Ambiguity x Observer JS interaction was significant (see Model 1b) and showed a similar pattern to the one observed in Studies 1-3 – supporting H2b’ (see Figure S6).

Next, we examined the subset of participants in the *resolve/not resolve* condition. Most chose to resolve the ambiguity (87.9%), whereas a minority did not (12.1%). The logistic regression model showed that this decision was not predicted by Observer JS, Wald (1) = 0.19, *p* = .849, OR = 1.05, 95% CI [0.65, 1.68], nor Perpetrator JS, Wald (1) = 1.49, *p* = .137, OR = 1.40, 95% CI [0.90, 2.17]; thus, H3 was not supported.

Furthermore, we compared the levels of 3PP across the different subsets of participants (see Figure S7). The first regression model showed those who resolved the ambiguity punished significantly more than those who did not – supporting H4; Dummy 1, β = .57, *t*(303) = 3.29, *p* = .001, 95% CI [.23, .91]. The second model showed that those who resolved the ambiguity did not punish more than those in the *no ambiguity* condition – not supporting H5; Dummy 2, β = -.11, *t*(429) = -1.07, *p* = .285, 95% CI [-.30, .09]. The third model showed that those who did not resolve the ambiguity did not punish less than those in the *ambiguity* condition – not supporting H6; Dummy 3, β = .04, *t*(200) = 0.24, *p* = .807, 95% CI [-.32, .40].

| **Table S12**  *Tested Multiple Regression Models on Punishment in Study 4.* | | | | | | | | |
| --- | --- | --- | --- | --- | --- | --- | --- | --- |
|  |  | **Model 1a (preregistered)** | | |  | **Model 1b** | | |
| **Parameters** |  | **β** [95% CI] | ***t*** | ***p*** |  | **β** [95% CI] | ***t*** | ***p*** |
| Ambiguity of Norm Violation |  | -.68 [-.88, -.48] | -6.65 | **< .001***** |  | -.68 [-.88, -.48] | -6.68 | **< .001***** |
| Perpetrator JS |  | .13 [-.05, .32] | 1.39 | .165 |  | **-** | **-** | **-** |
| Observer JS |  | .20 [.02, .38] | 2.14 | **.033*** |  | .28 [.14, .42] | 3.87 | **< .001***** |
| Ambiguity x Perpetrator JS |  | -.08 [-.33, .17] | -0.61 | .545 |  | **-** | **-** | **-** |
| Ambiguity x Observer JS |  | -.21 [-.45, .04] | -1.67 | .097 |  | -.26 [-.46, -.06] | -2.57 | **.011 *** |
| Perpetrator JS x Observer JS |  | **-** | **-** | **-** |  | **-** | **-** | **-** |
| Ambiguity x Perpetrator JS x Observer JS |  | **-** | **-** | **-** |  | **-** | **-** | **-** |
| Observations |  | 328 | | |  | 328 | | |
| R^2^ / Adj. R^2^ |  | .176 / .163 | | |  | .170 / .162 | | |
| *Note*. *** *p* < .001, ** *p* < .01, * *p* < .05. | | | | | | | | |

**
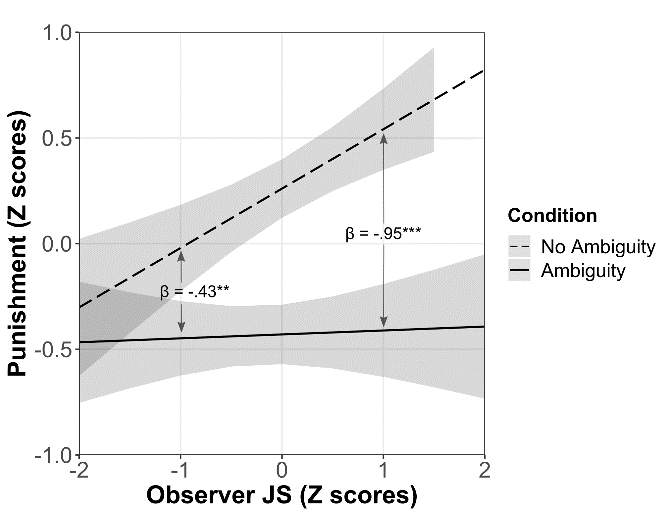
**

**Figure S6**

*Two-way interaction between Ambiguity and Observer JS in Study 4. Band widths 95% CIs.*

**Figure S7**

*Levels of 3PP in experimental conditions and self-selected groups in Study 4. 95% CIs error bars.*


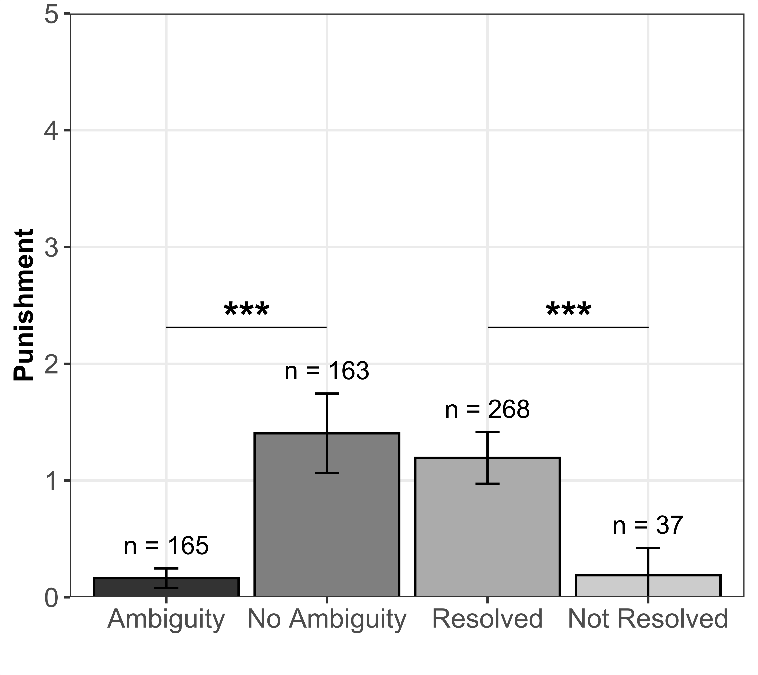


**Exploratory Results**

In the post-experimental questionnaire, we observed that participants who resolved the ambiguity reported significantly higher concerns about making an unfair decision and significantly lower concerns about avoiding costs than those who did not resolve the ambiguity (Figure S8 A). Furthermore, the former reported a drastically higher sense of curiosity.

Regarding the punishment decision (Figure S8 B), we observed that, in the *ambiguity* condition, participants reported significantly higher concerns about making an unfair decision than those in the *no ambiguity* condition and those who resolved or kept the ambiguity in the *resolve/not resolve* condition. We did not observe significant differences in cost avoidance.

We also explored whether type I error concerns and cost avoidance mediated the effect of ambiguity on 3PP (see Figure S9). As in Study 4, we only used the data from the ambiguity and the no ambiguity conditions. The mediation model showed that ambiguity was positively associated with type I error concerns but not cost avoidance. In contrast to Study 4, type I error concerns and cost avoidance negatively predicted 3PP. The indirect effect through type I error concerns was significant, *a_1_b_1_* = -0.11, 95% CI [-0.21, -0.02], but the indirect effect through cost avoidance was not, *a_2_b_2_* = -0.08, 95% CI [-0.26, 0.09] (bootstrap 5000 iterations).

**Figure S8**

*Differences in Types of Concerns Associated with the Decision to Resolve the Ambiguity (A) and the Decision to Punish (B) in Study 4b.*


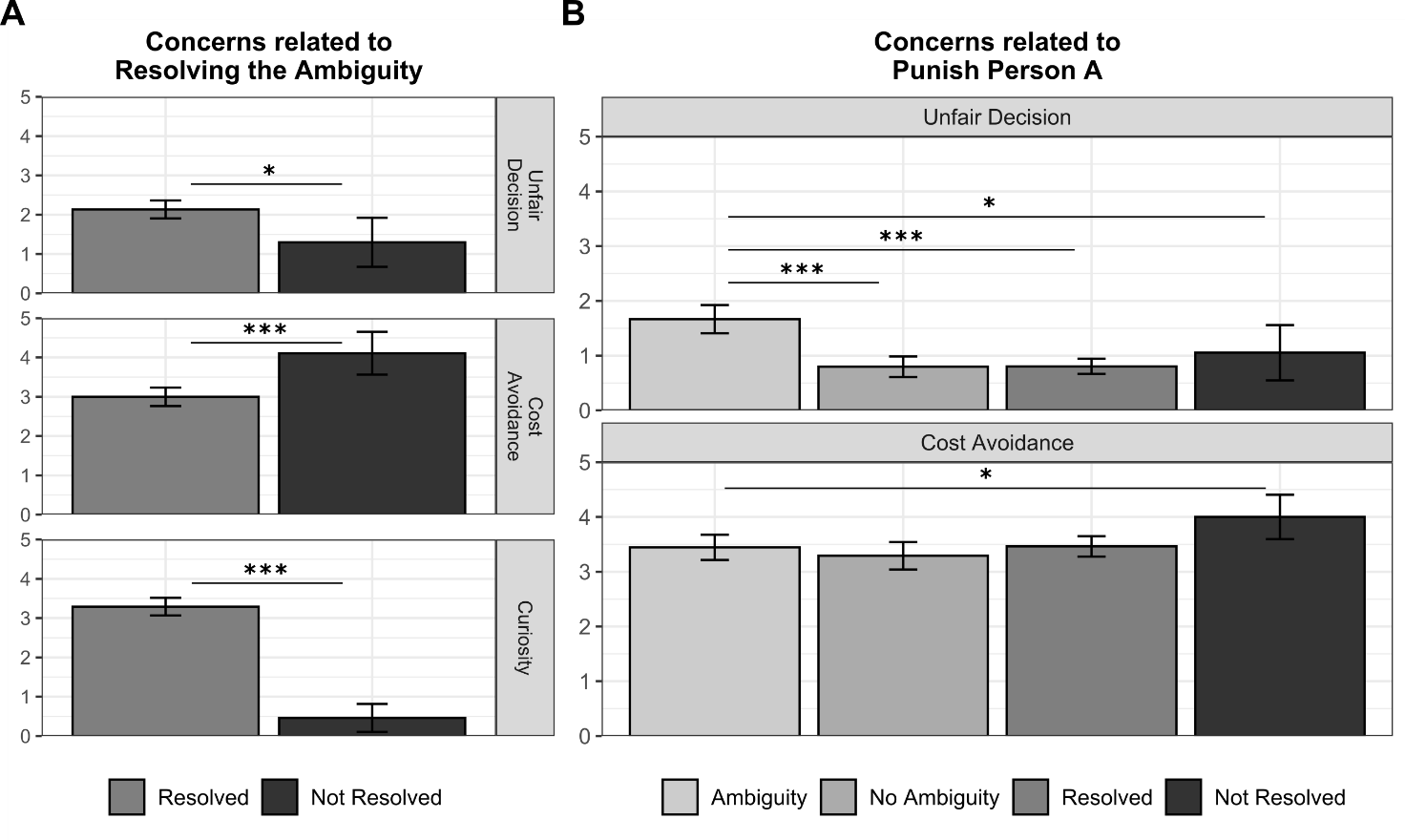


*Note*. *p*-values correspond to Welch independent sample t-tests (A) and linear regressions including Condition as dummy-coded predictor (B).

*** *p* < .001, ** *p* < .01, * *p* < .05. 95% CIs error bars.

**Figure S9**


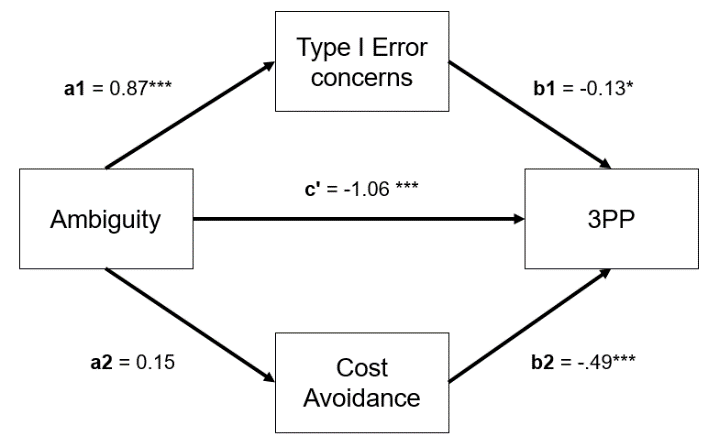
*Exploratory Parallel Mediational Model Tested in Study 4b.*

*Note.* *** *p* < .001, ** *p* < .01.

**Discussion**

In Study 4b, we replicated the negative effect of ambiguity on 3PP and the moderating role of Observer JS (in the model without Perpetrator JS).

Similarly to Study 4, Study 4b suggested that ambiguity induced type I error concerns that potentially hindered 3PP. Those who had the opportunity, and decided to, resolve the ambiguity subsequently punished to similar extents than those in the *no ambiguity* condition. This indicated that resolving the ambiguity could have alleviated the concerns that otherwise made participants hesitate to punish. In fact, results from the post-experimental questionnaire supported our argument by showing that such concerns significantly mediated the effect of ambiguity on 3PP. Under ambiguity, participants reported higher type I error concerns, and these significantly decreased 3PP.

In contrast to Study 4, a greater proportion of participants resolved the ambiguity, presumably because this option was cost-free and, as the post-experimental questionnaire clearly indicated, some people resolved the ambiguity out of mere curiosity. Thus, among participants who resolved the ambiguity, it was difficult to differentiate between those who intended to inform their decision to react against the (potential) norm violation and those who were merely curious. This issue could explain why those who resolved the ambiguity did not show higher 3PP than the baseline 3PP observed under no ambiguity, but it was higher than the 3PP observed under ambiguity. Due to the previous, we decided to make the decision of resolving the ambiguity costly in Study 4. As discussed in the main manuscript, we expected that introducing a cost would discourage third parties who resolved ambiguity merely out of curiosity from doing so.

Furthermore, there was another difference with Study 4, namely that the Ambiguity x Observer JS interaction was significant. It is unclear why this effect did not similarly replicate across studies, given the similarities in their design, procedure and measurement of JS. For Study 4b, however, we recruited a younger sample of undergraduate students (vs. general population sample in Study 4), which showed similar average levels of Observer JS but lower 3PP across conditions. It is possible that these demographics moderated the effect, but at present, we cannot offer a theoretically-grounded explanation for why this might be the case.

# Power Analyses

## Study 3

For Study 3, we conducted a priori power simulations to plan our data collection. Specifically, we used the standardized estimates observed in Study 1 to generate random multilevel data (1000 iterations). Specifically, these estimates corresponded to a simplified multilevel model including the effects of ambiguity of the norm violation, Observer JS and their two-way interaction. We assumed Observer JS to be normally distributed and a critical α = .05. The results of the simulations indicated that the targeted sample size of 300 participants would suffice to detect a significant Ambiguity x Observer JS with 95.1% statistical power (see Figure S10). The script for reproducing this simulation is available in the general [OSF repository](https://osf.io/2q9vm/) linked to the main manuscript (i.e., “Study 1/4. Power Simulation for Study 3.R”)

**Figure S10**


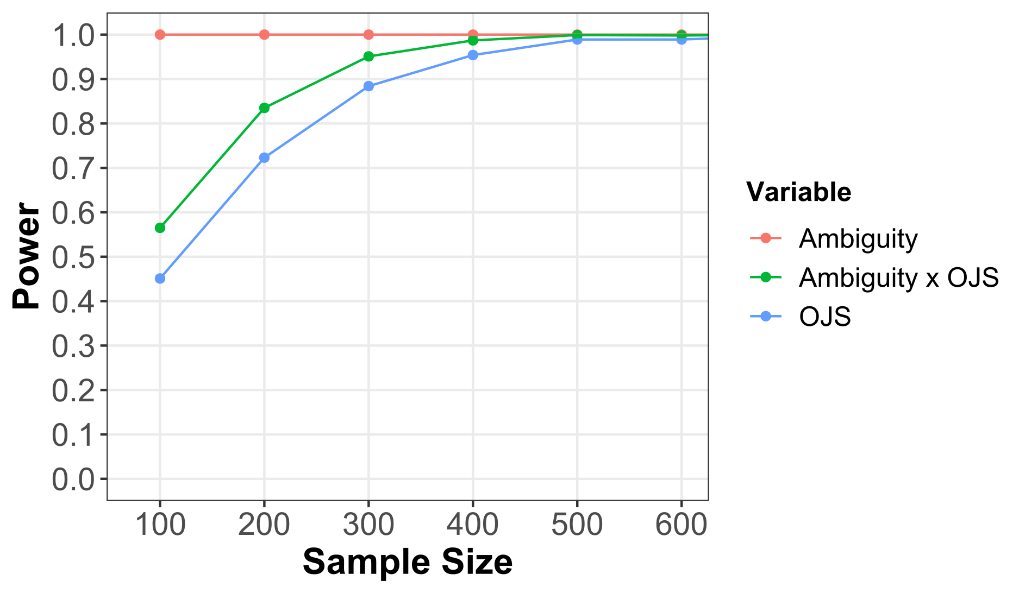
*Power Curve for Effects of Ambiguity, Observer JS, and Their Two-Way Interaction.*

## Studies 4 and 4b

In our preregistration, we described an a priori sample size estimation uniquely based on the ambiguity effect observed in Study 3. Since the design of Studies 4 and 4b did not make use of the strategy method, we took as a reference the between-subject difference between the no ambiguity and ambiguity conditions (i.e., using only the first round of the 3PPG) for the decision of the strategy method in which the dictator distributed 1 ECU (i.e., *d* = -66). A sample size of *N* = 102 already guaranteed to detect this effect size with 95% statistical power in a one-tailed t-test, which we originally preregistered.

However, before beginning the data collection, we considered a second power estimation to guarantee the potential replication of the Ambiguity x JS interaction. We used as reference the significant Ambiguity x Perpetrator JS interaction that we observed for the single decision of distributing 1 ECU in Study 3 (β = .297). A sample size of *N* = 300 would suffice to detect this interaction effect with 80% statistical power.

As indicated in the main manuscript, we additionally planned to compare the no ambiguity and ambiguity conditions with the subset of participants who decided (not) to resolve the ambiguity in our third experimental condition. Thus, we planned to collect double the sample size (*N* = 600) to balance the number of participants in each compared group.

For the power estimations, we used the R packages “pwr” (version 1.3-0., Champely, 2020) and “pwr2ppl” (version 0.1.2, Aberson, 2019), respectively.

# Elicitation of Dictator Decisions in Studies 4, 4b and 5

Studies 4, 4b and 5 followed a two-stage procedure. In the first stage, we recruited a small group of participants who made decisions in the roles of Person A and Person B (n = 11 in Study 4, n = 20 in Study 4b, and n = 29 in Study 5). The second stage was the actual experiment, where we collected the reactions from Person Cs (for a similar procedure, see Kurzban et al., 2007).

In the first stage, we elicited the targeted norm transgression (i.e., distributions of 1 ECU from an endowment of 10 ECUs). In order to do so, we simplified Person A’s decision to a dichotomous choice between a fair distribution (i.e., 5 ECUs – 5 ECUs) and an unfair distribution (i.e., 1 ECU – 9 ECUs). Note that in the conditions involving ambiguity of the norm violation, Person A’s endowment was randomly determined between 2 and 10 ECUs. If the endowment was different from 10 ECUs, it would not allow a clean comparison with the no ambiguity condition. Therefore, in order to determine random endowments while assuring that most of them were 10 ECUs, we tweaked the underlying probability distribution, so that only in 10% of the cases Person A would receive an amount different from 10 ECUs. We informed participants in the main experiments that Person A’s endowment was determined according to a probability distribution that was unknown to them.

Once we collected the decisions, we formed dyads and randomly assigned participants to the role of Person A and Person B. Those dyads in which Person A distributed fairly or Person A received an endowment different from 10 ECUs were paid according to Person A’s decision. In contrast, for those dyads in which Person A decided on the unfair distribution, we applied the average punishment and compensation in each experimental condition as participants in the role of Person C would later decide in the second stage.

# Post-Experimental Questionnaire – Studies 4, 4b and 5

## Complete List of Items Presented in each Study

**Table S13**

*List of Items Presented in the Post-Experimental Questionnaire of each Study.*

| **Items** | **Study 4** | **Study 4b** | **Study 5** |
| --- | --- | --- | --- |
| **Concerns Related to Resolving Ambiguity** |  |  |  |
| PQ_R_1. I've been thinking about the risk of being unfair to person A. | ✓ | ✓ | - |
| PQ_R_2. I made my decision out of curiosity. | ✓ | ✓ | - |
| PQ_R_3. I felt responsible for what happened between person A and person B. | ✓ | ✓ | - |
| PQ_R_4. My priority was to avoid costs. | ✓ | ✓ | - |
| PQ_R_5. I decided in a way not to feel bad. | ✓ | - | - |
| PQ_R_6. It was important to me that I would not have negative feelings about my decision regarding Person A. | ✓ | - | - |
| PQ_R_7. I definitely wanted to avoid making an unfair decision | ✓ | - | - |
| **Concerns Related to Punishment** |  |  |  |
| PQ_1. I was concerned that my decision about person A could be unfair. | ✓ | ✓ | ✓ |
| PQ_2. I wanted to avoid being unfair to person A. | ✓ | ✓ | - |
| PQ_3. I was concerned that Person A might see me as an unfair person based on my decision. | ✓ | ✓ | ✓  (relabeled as PQ_2) |
| PQ_4. I was concerned with whether person A could perceive me as mean. | ✓ | ✓ | ✓  (relabel as PQ_3) |
| PQ_5. I was concerned about feeling like a malefactor. | ✓ | ✓ | ✓  (relabeled as PQ_4) |
| PQ_6. I would feel bad myself if my decision had been unfair. | ✓ | ✓ | - |
| PQ_7. I felt that I didn't have enough information to make my decision. | ✓ | PQ_7 was “I felt that my decision was not informed”. | ✓  (relabeled as PQ_5) |
| PQ_8. I had all the information I needed to make the decision. **(R)** | ✓ | ✓ | ✓  (relabeled as PQ_6R) |
| PQ_9. I thought that what happened between person A and person B had nothing to do with me. **(R)** | ✓ | ✓ | - |
| PQ_10. I felt responsible for what happened between person A and person B. | ✓ | ✓ | - |
| PQ_11. I intended to split the points between person A and person B more fairly. | ✓ | ✓ | ✓  (relabeled as PQ_9) |
| PQ_12. I ideally wanted person A and person B to receive the same number of points. | ✓ | ✓ | ✓  (relabeled as PQ_10) |
| PQ_13. It was my intention to make person A understand that they should change their behavior in the future. | ✓ | ✓ | - |
| PQ_14. I was hoping that person A took my decision seriously and improved her behavior. | ✓ | ✓ | - |
| PQ_15. I did what I thought Person B wanted. | ✓ | ✓ | - |
| PQ_16. I then decided what I would have wanted in person B's place. | ✓ | ✓ | - |
| PQ_17. My priority was to avoid costs. | ✓ | ✓ | ✓  (relabeled as PQ_7) |
| PQ_18. I have barely taken into account the cost of my decision. **(R)** | ✓ | ✓ | ✓  (relabeled as PQ_8R) |
| PQ_19. I did not want to feel as if I had been mean against Person A. | ✓ | - | - |
| PQ_20. I thought I would feel bad if I had been unfair against Person A. | ✓ | - | - |

*Note*. Those items marked with ✓ were included in the respective studies. Those items shaded in gray were not entered in Principal Component Analyses (PCA) for consistency purposes with the other studies.

## Bivariate Correlations between Post-Experimental Questionnaire, JS, and SVO

**Table S14**

*Bivariate Correlations between Post-Experimental Questionnaire Dimensions and JS scales – Study 4.*

| Variable | 1 | 2 | 3 |
| --- | --- | --- | --- |
| **Post-Experimental Questionnaire** |  |  |  |
| 1. Unfair Decision |  |  |  |
| 2. Cost Avoidance | -.12**  [-.19, -.05] |  |  |
| **Justice Sensitivity** |  |  |  |
| 3. Observer JS | .26**  [.19, .33] | -.13**  [-.20, -.06] |  |
| 4. Perpetrator JS | .27**  [.20, .33] | -.20**  [-.27, -.13] | .55**  [.50, .60] |

*Note.* Values in brackets indicate the 95% CI for each correlation. *** *p* < .001.

**Table S15**

*Bivariate Correlations between Post-Experimental Questionnaire Dimensions and JS scales – Study 4b.*

| Variable | 1 | 2 | 3 |
| --- | --- | --- | --- |
| **Post-Experimental Questionnaire** |  |  |  |
| 1. Unfair Decision |  |  |  |
| 2. Cost Avoidance | -.17**  [-.24, -.09] |  |  |
| **Justice Sensitivity** |  |  |  |
| 3. Observer JS | .11**  [.03, .18] | -.22**  [-.29, -.15] |  |
| 4. Perpetrator JS | .10**  [.03, .18] | -.22**  [-.30, -.15] | .62**  [.57, .67] |

*Note.* Values in brackets indicate the 95% CI for each correlation. *** *p* < .001.

**Table S16**

*Bivariate Correlations between Post-Experimental Questionnaire Dimensions and JS scales – Study 5.*

| Variable | 1 | 2 | 3 |
| --- | --- | --- | --- |
| **Post-Experimental Questionnaire** |  |  |  |
| 1. Unfair Decision |  |  |  |
| 2. Cost Avoidance | -.12**  [-.18, -.05] |  |  |
| **Justice Sensitivity** |  |  |  |
| 3. Observer JS | .11**  [.04, .18] | -.17**  [-.24, -.10] |  |
| 4. Perpetrator JS | .16**  [.09, .23] | -.17**  [-.23, -.10] | .52**  [.46, .57] |

*Note.* Values in brackets indicate the 95% CI for each correlation. *** *p* < .001.

## Participants’ Assumptions about Person A’s Endowment under Ambiguity

**Figure S11**

*Percentages of Reported Assumptions about Person A’s Endowment in Studies 4 (A) and 4b (B).*


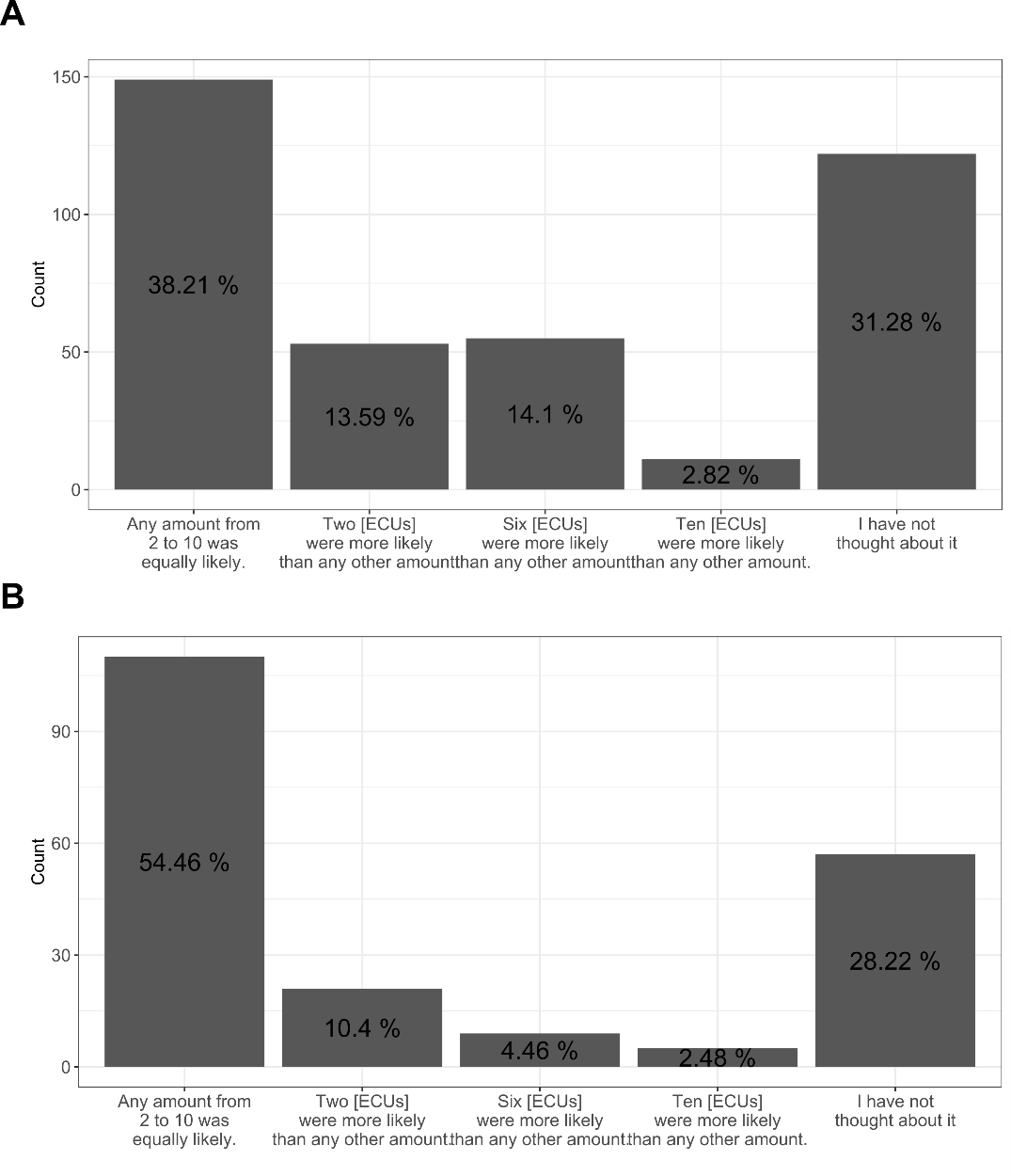


*Note.* The figure presents the percentage from the total number of participants who remained ignorant about Person A’s endowment (i.e., those in the *ambiguity* condition and those who did *not resolve* ambiguity).

# Study 5 – Secondary Analyses

## Analyses with Preregistered Items of Type I Error

As indicated in the preregistration of Study 5, we planned to measure type I error concerns through a scale consisting of 4 items, which intended to capture different aspects that could characterize this type of concern (e.g., concerns related to unfair punishment, social image, and subjective negative feelings). Participants expressed their agreement with the following items (relabeled PQ_1, PQ_2, PQ_3 and PQ_4, see Table S13 for the complete post-experimental questionnaire) using a 6-point Likert scale (0 – *Not at all*, 5 – *Extremely*):

PQ_1 *“I was concerned that my decision about Person A could be unfair.”*PQ_2 *“I was concerned that Person A might see me as an unfair person based on my decision.”*
PQ_3 *“I was concerned with whether person A could perceive me as mean.”*
PQ_4 *“I was concerned about feeling like a malefactor.”*

The scale showed high internal consistency (α = .86) and we computed an aggregate score.

The results from the mediational model were not different from those reported in the main manuscript. Ambiguity significantly predicted type I error concerns and 3PP, however, type I error concerns did not predict 3PP (Figure S12). The indirect effect of ambiguity on 3PP through type I error concerns was not significant, *ab =* 0.00, 95% CI [-0.05, 0.06] (bootstrap 5000 iterations).

**Figure S12**
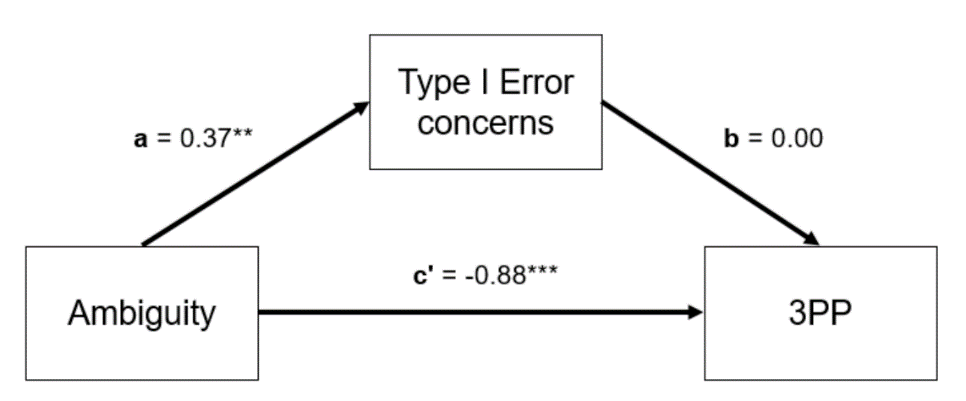
*Preregistered Mediation Model Tested in Study 5.*

*Note*. *** *p* < .001, ** *p* < .01.

## SVO and Inequality Aversion

In Study 5, we assessed Social Value Orientation (SVO; van Lange et al., 1997) as an additional validated measure of fairness concerns, to explore whether social preferences and, specifically, inequality aversion played a similar role under ambiguity to the one JS arguably played.

We measured SVO using the SVO slider measure (Murphy et al., 2011), which consists of 15 items. In this study, the distributed points had a real monetary value of €0.01. At the end of the study, we grouped participants in dyads, we randomly assigned each dyad’s member to the role of giver or receiver, and we paid them according to one of the 15 decisions selected at random. We used the nine secondary items to calculate an *index of inequality aversion* (Murphy et al., 2011), which captured the average normalized difference between participants’ choices and the option that maximized equality. Thus, low values of this index indicated high inequality aversion. Figure S13 offers information about the distribution of the SVO angle measure and the index of inequality aversion.

**Figure S13**

*Distribution of SVO Angles (A) and Inequality Aversion Index (B) from SVO Slider Measure.*

**A**


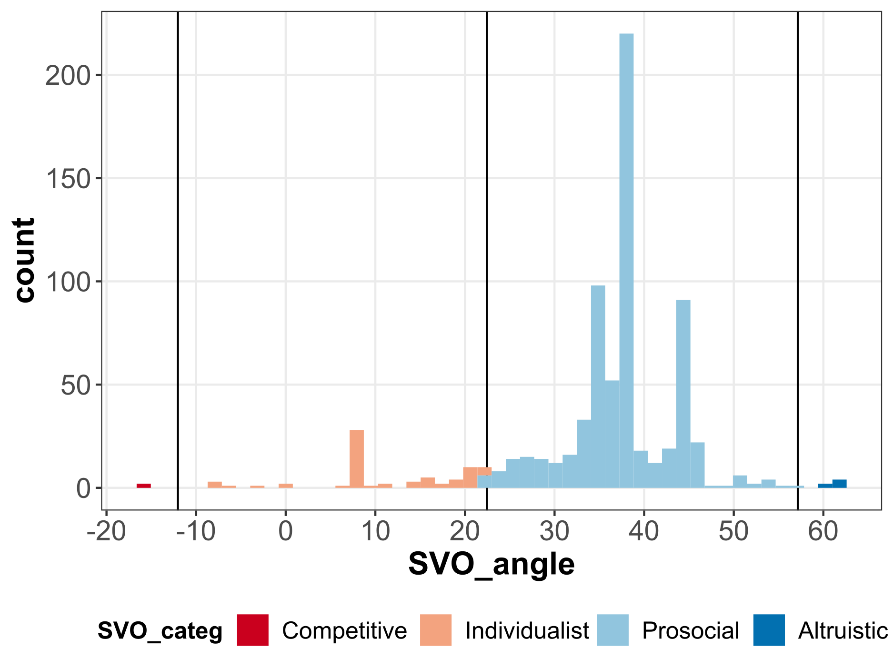


**B**


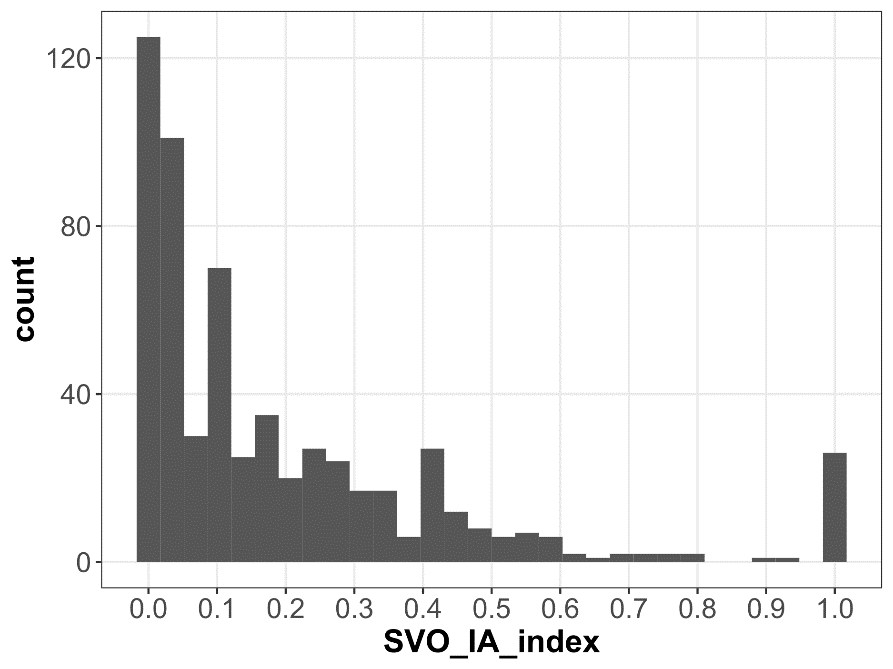


As summarized in Tables S17 and S18 (see p. 34), Observer JS and SVO moderated neither the effect of ambiguity (even when only considering the *high expected value* conditions) nor the effect of expected value.

## OJS and SVO interactions with Ambiguity and Expected Value.

| **Table S17**  *Exploratory multiple regression model to check if Observer JS moderated the effects of Ambiguity and Expected Value on Punishment in Study 5.* | | | | |
| --- | --- | --- | --- | --- |
| **Parameters** | **β** [95% CI] | | ***t*** | ***p*** |
| Ambiguity of Norm Violation | .02 [-.18, .21] | | 0.16 | .870 |
| Expected Value | .76 [.56, .95] | | 7.66 | < .001*** |
| Observer JS | .08 [-.07, .22] | | 1.04 | .299 |
| Ambiguity x Expected Value | -.57 [-.84, -.30] | | -4.10 | < .001*** |
| Ambiguity x Observer JS | -.15 [-.35, .05] | | -1.47 | .141 |
| Expected Value x Observer JS | .05 [-.14, .24] | | 0.50 | .614 |
| Ambiguity x Expected Value x Observer JS | .13 [-.15, .40] | | 0.92 | .359 |
| Observations |  | 755 | | |
| R^2^ / Adj. R^2^ |  | .100 / .092 | | |
| *Note*. *** *p* < .001, ** *p* < .01, * *p* < .05. | | | | |

| **Table S18**  *Exploratory multiple regression model to check if the SVO Inequality Aversion index moderated the effects of Ambiguity and Expected Value on Punishment in Study 5.* | | | | |
| --- | --- | --- | --- | --- |
| **Parameters** | **β** [95% CI] | | ***t*** | ***p*** |
| Ambiguity of Norm Violation | .01 [-.18, .21] | | 0.10 | .918 |
| Expected Value | .71 [.51, .91] | | 7.12 | < .001*** |
| SVO_IA_index | .00 [-.13, .14] | | 0.09 | .927 |
| Ambiguity x Expected Value | -.53 [-.81, -.25] | | -3.77 | < .001*** |
| Ambiguity x Observer JS | .04 [-.15, .23] | | 0.39 | .700 |
| Expected Value x Observer JS | -.14 [-.35, .06] | | -1.37 | .172 |
| Ambiguity x Expected Value x Observer JS | .12 [-.16, .40] | | 0.84 | .399 |
| Observations |  | 741 | | |
| R^2^ / Adj. R^2^ |  | .092 / .084 | | |
| *Note*. *** *p* < .001, ** *p* < .01, * *p* < .05. | | | | |

# References

Aberson, C. L. (2019). *Applied power analysis for the behavioral sciences* (2nd ed.). Routledge. https://doi.org/10.4324/9781315171500

Barberis, N. (2013). The psychology of tail events: Progress and challenges. *American Economic Review*, *103*(3), 611–616. https://doi.org/10.1257/aer.103.3.611

Baumert, A., & Schmitt, M. (2016). Justice sensitivity. In C. Sabbagh & M. Schmitt (Eds.), *Handbook of Social Justice Theory and Research* (pp. 161–180). Springer New York. https://doi.org/10.1007/978-1-4939-3216-0_9

Champely, S. (2020). *pwr: Basic functions for power analysis* (version 1.3-0.) [Computer software]. https://CRAN.R-project.org/package=pwr

Egas, M., & Riedl, A. (2008). The economics of altruistic punishment and the maintenance of cooperation. *Proceedings of the Royal Society B: Biological Sciences*, *275*(1637), 871–878. https://doi.org/10.1098/rspb.2007.1558

Kurzban, R., Descioli, P., & Obrien, E. (2007). Audience effects on moralistic punishment. *Evolution and Human Behavior*, *28*(2), 75–84. https://doi.org/10.1016/j.evolhumbehav.2006.06.001

Lichtenstein, S., Slovic, P., Fischhoff, B., Layman, M., & Combs, B. (1978). Judged frequency of lethal events. *Journal of Experimental Psychology: Human Learning and Memory*, *4*(6), 551–578. https://doi.org/10.1037/0278-7393.4.6.551

Rozin, P., & Royzman, E. B. (2001). Negativity bias, negativity dominance, and contagion. *Personality and Social Psychology Review*, *5*(4), 296–320. https://doi.org/10.1207/S15327957PSPR0504_2

Tversky, A., & Kahneman, D. (1992). Advances in prospect theory: Cumulative representation of uncertainty. *Journal of Risk and Uncertainty*, *5*(4), 297–323. https://doi.org/10.1007/BF00122574
